# Distinct strategies of diguanylate cyclase domain proteins on inhibition of virulence and interbacterial competition by agrobacteria

**DOI:** 10.1128/mbio.00039-25

**Published:** 2025-04-17

**Authors:** Xuan Lai, Manda Yu, Chiu-Ping Cheng, Erh-Min Lai

**Affiliations:** 1 Institute of Plant and Microbial Biology, Academia Sinica 71559, Taipei, Taiwan; 2 Institute of Plant Biology, National Taiwan University 124717 , Taipei, Taiwan; 3 Institute of Plant Pathology and Microbiology, National Taiwan University 33561, Taipei, Taiwan; National University of Singapore, Singapore, Singapore

**Keywords:** diguanylate cyclases, c-di-GMP, virulence regulation, interbacterial competition, *Agrobacterium tumefaciens*

## Abstract

Diguanylate cyclases (DGCs) synthesize bis-(3′,5′)-cyclic diguanylic acid (c-di-GMP), a critical bacterial second messenger that coordinates diverse biological processes. *Agrobacterium tumefaciens*, a plant pathogen causing crown gall disease, relies on type IV secretion system for pathogenesis and type VI secretion system (T6SS) for interbacterial competition. Our study identified two putative DGCs, named diguanylate cyclase domain proteins regulating virulences A and B (DcvA and DcvB), that negatively regulate virulence through distinct mechanisms. DcvA suppresses virulence by targeting the VirA/VirG two-component system downstream of VirA. This inhibition is independent of c-di-GMP levels. DcvB positively regulates biofilm formation, inhibits T6SS-mediated interbacterial competition, and suppresses virulence via the ChvG/ChvI two-component system downstream of ChvG. These effects are dependent on its cyclase activity and the associated increase in intracellular c-di-GMP levels. These findings suggest that DcvA and DcvB control virulence and interbacterial competition using different mechanisms in *Agrobacterium*. DcvA suppresses virulence, independent of c-di-GMP, and DcvB enhances global c-di-GMP concentration to promote biofilm formation and inhibits virulence and T6SS antibacterial activity. The findings provide understanding of how DGC domain proteins orchestrate complex regulatory networks to balance virulence, biofilm formation, and interbacterial competition, enabling them to adapt to changing environments.

**IMPORTANCE:**

Bacteria produce second messengers, such as c-di-GMP, to regulate various cellular processes, including biofilm formation, virulence, and bacterial antagonism. Diguanylate cyclases (DGCs) catalyze the biosynthesis of c-di-GMP and function to cope with changing environments through targeting specific effector proteins. In this study, we uncover that phytopathogenic agrobacteria deploy two DGC domain proteins to suppress virulence and interbacterial competition through two different regulatory pathways. One exhibits the DGC activity, enhancing global c-di-GMP concentration to elevate biofilm formation and inhibit virulence and antibacterial activity, while the other specifically suppresses virulence, independent of c-di-GMP biosynthesis. Our findings provide new insight into the distinct regulatory mechanisms of DGC domain proteins on regulating virulence and interbacterial competition, highlighting potential new strategies for controlling *Agrobacterium* pathogenicity.

## INTRODUCTION

Bis-(3′,5′)-cyclic diguanylic acid (c-di-GMP) is a nucleotide-based second messenger predominantly found in prokaryotes (1). c-di-GMP can be synthesized from two guanosine triphosphate molecules by diguanylate cyclases (DGCs), which contain a conserved GGDEF (or GGEEF) domain. In addition to DGCs, c-di-GMP-specific phosphodiesterases (PDEs) hydrolyze c-di-GMP through direct binding (2). DGC transduces signals to effector proteins for regulating various cellular processes, including biofilm formation, motility, virulence, and interbacterial competition (2, 3). Swimming motility driven by bacterial flagella is suppressed by c-di-GMP (4), while biofilm formation involving the production of exopolysaccharide for surface attachment is promoted by c-di-GMP (5, 6). c-di-GMP can regulate virulence by targeting secretion systems in plant pathogens (7). For example, c-di-GMP regulates the type II secretion system (T2SS)-secreted cell wall degradation enzymes in *Dickeya dadantii* that causes soft rot disease. The DGC GcpA inhibits the Rsm small RNA regulatory system to downregulate the expression of T2SS (8). The two-component system CpxA/CpxR controls the expression of the type III secretion system (T3SS) through the manipulation of genes affecting the c-di-GMP turnover (9). Another example in olive knot pathogen *Pseudomonas savastanoi* pv. *savastanoi*, DgcP produced c-di-GMP has a positive role in regulating virulence gene expression and type VI secretion system (T6SS) but not T3SS (10).

Members of the *Agrobacterium* genus are capable of causing crown gall or hairy root disease on plants (11–13). Agrobacteria are soil inhabitants that can sense plant signals to swim toward the host for colonization and form biofilms on its surface (14, 15). Plant phenolic compounds (e.g., acetosyringone [AS]) released from the plant wounds are sensed by the VirA/VirG two-component system for virulence induction (16, 17). The detection of phenolics by sensor kinase VirA leads to the phosphorylation and activation of response regulator VirG (18, 19). Phosphorylated VirG binds to the promoter region of virulence genes (*vir*) encoded on the tumor-inducing plasmid (Ti plasmid) and activates their transcription (20, 21). The Vir proteins facilitate the cleavage of transfer DNA (T-DNA) and form a type IV secretion system (T4SS) composed of the VirB/VirD4 transmembrane protein channel (22). T-DNA and effectors, such as VirE2, are transferred through the T4SS from *Agrobacterium* cells into the plant cell, ultimately leading to tumorigenesis (17, 23, 24).

The regulation of *Agrobacterium* virulence is coordinated with interbacterial competition (25). The T6SS is a nanomachine that delivers effectors for interbacterial competition, metal acquisition, or pathogenesis in eukaryotic hosts (26). In *Agrobacterium*, the T6SS functions as an antibacterial weapon, providing competitive growth advantages *in vitro* and *in planta* (27, 28), as well as promoting disease (29). The T6SS gene cluster consisting of the *imp* and *hcp* operons is prevalent and highly conserved in the *Agrobacterium* species complex (30, 31). The *imp* operon encodes the structural components (TssA-M) necessary for assembling membrane-associated contractile T6SS machinery, and the *hcp* operon encodes effectors and Hcp-VgrG tail-tube modules for delivery (32).

*Agrobacterium* virulence and interbacterial competition are also regulated by the pH of the environment via the ExoR-ChvG/ChvI regulatory system. Under neutral pH conditions, the periplasmic repressor ExoR binds to and inhibits the activation of the sensor kinase ChvG. Upon sensing acidic signal, ExoR is rapidly degraded, derepressing ChvG, which then phosphorylates the response regulator ChvI (25). Phosphorylated ChvI binds to the second promoter of *virG*, enhancing its transcription (33–35). The cooperation of ChvG/ChvI with VirA/VirG maximizes *vir* gene induction. Phosphorylated ChvI also binds to the divergent promoters of *imp* and *hcp* operons, activating the transcription of T6SS genes (25).

The genome of *Agrobacterium* strain C58 encodes 16 *dgc* genes, 13 *dgc-pde* genes, and two *pde* genes (15). Several *Agrobacterium* DGCs positively regulate biofilm formation by increasing intracellular c-di-GMP levels without affecting virulence. However, few DGCs, such as PleD (also named as CelR, Atu1297), severely attenuate the virulence (36, 37). Interestingly, overexpressing the exogenous transmembrane-DGC (TM-DGC) SadC from *Pseudomonas aeruginosa* PAO1 inhibits the expression of both *vir* and T6SS genes in *Agrobacterium* C58, leading to reduced antibacterial activity and transient transformation efficiency (38). Further overexpression of six endogenous *Agrobacterium* TM-DGCs found that three of them negatively regulate T6SS, but their roles in regulating virulence and other biological processes are not fully explored.

Based on the findings that six TM-DGCs have varying effects on T6SS and *vir* gene expression (38), we hypothesized that different *Agrobacterium* TM-DGCs may regulate interbacterial competition and virulence through distinct mechanisms by interfering with regulatory components. Among them, Atu5372 negatively regulates both T6SS and *vir* genes, while Atu2691 has no detectable impact on either process (38). In addition to Atu2691, overexpression of Atu1207 also does not affect T6SS; however, its role in virulence regulation remains unknown (38). Given our objective to investigate the regulatory mechanisms of the TM-DGCs involved in virulence, we focus on studying Atu5372 and aim to identify a TM-DGC that specifically influences virulence without affecting T6SS. Atu1207 emerges as a promising candidate.

This study reveals the regulatory roles of two TM-DGCs, Atu1207 and Atu5372, hereafter referred to as DcvA and DcvB (diguanylate cyclase domain proteins regulating virulences A and B), respectively, in *Agrobacterium* strain C58. By employing a combination of gene regulation analyses, c-di-GMP measurements, and various phenotypic assessments of deletion mutants and overexpression strains, we demonstrate that DcvA and DcvB govern *Agrobacterium* virulence and T6SS-mediated interbacterial competition through distinct mechanisms. These findings underscore their critical roles in shaping *Agrobacterium* adaptation in niche competition and plant infection strategies.

## RESULTS

### Two DGC domain-containing proteins negatively regulate transient transformation and virulence in *Agrobacterium* C58

**Fig 1:**
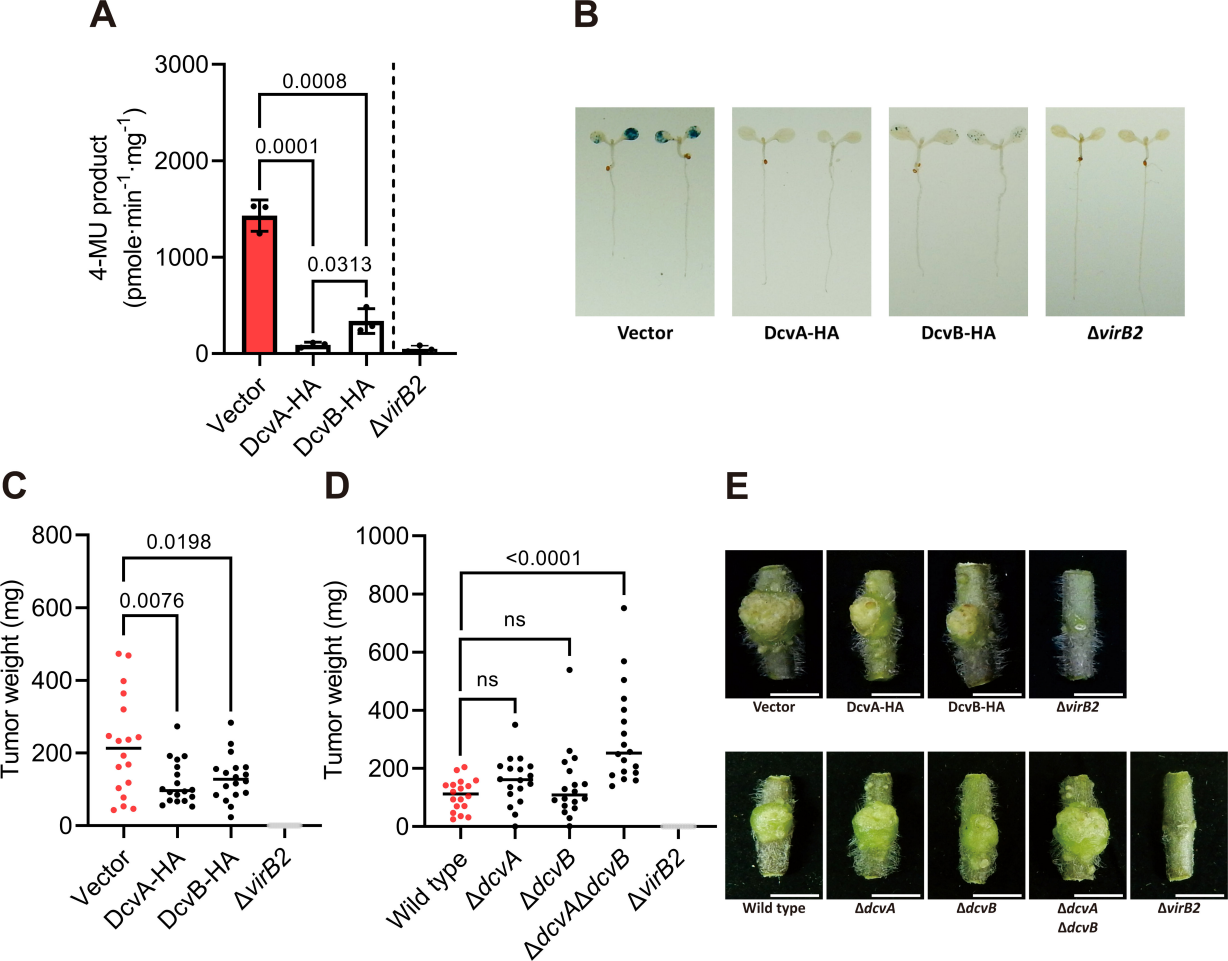
DcvA and DcvB negatively regulate transformation and virulence. (A) Transient transformation efficiency determined by the quantitative GUS activity with overexpression strains carrying the GUS-intron reporter plasmid pBISN1. The Δ*virB2* mutant is a nonpathogenic mutant. Each dot represents a biological replicate, with each replicate consisting of 10 infected seedlings. Data are one representative result with mean ± standard deviation of three biological replicates. (B) Representative GUS staining of *A. thaliana* Col-0 seedlings. (C, D) Virulence assay measuring the tumor weight based on the tumors induced on wounded tomato stems with overexpression strains or deletion mutants. Each dot represents the weight of each tumor (*n* = 18), and the horizontal line indicates the median. Comparable results were observed in at least two independent experiments. The number atop each bar represents the *P*-value of the one-way analysis of variance with Tukey’s HSD test obtained by comparing to the vector control or wild-type C58. The term “ns” denotes no significant difference (*P* > 0.05). (E) Photographs of representative tumors. The scale bars represent 1 cm.

To investigate the roles of DcvA and DcvB in regulating virulence, we overexpressed HA-tagged DcvA and DcvB in *Agrobacterium* C58 (Fig. S1) to assess their impact on plant transformation and virulence. Compared to the wild-type C58 strain, we observed that both DcvA and DcvB significantly reduced transient transformation in *Arabidopsis thaliana* seedlings (Fig. 1A and B). Additionally, infection by strains overexpressing DcvA or DcvB resulted in reduced tumor size and weight on tomato stems relative to those caused by the wild-type C58 strain (Fig. 1C and E).

To further evaluate their roles, single and double in-frame deletion mutants were generated to examine their phenotypes. Results showed that neither Δ*dcvA* nor Δ*dcvB* single mutants showed significant effects on tumor weight. However, the double deletion mutant Δ*dcvA*Δ*dcvB* caused a significant increase in tumor weight (Fig. 1D and E). Interestingly, the double mutant was not associated with a significant increase in the relative transient transformation (Fig. S2).

These findings demonstrate that both TM-DGCs, DcvA and DcvB, play a negative regulatory role in the virulence of *Agrobacterium* C58.

### DcvA and DcvB downregulate the transcription of *vir* genes

**Fig 2:**
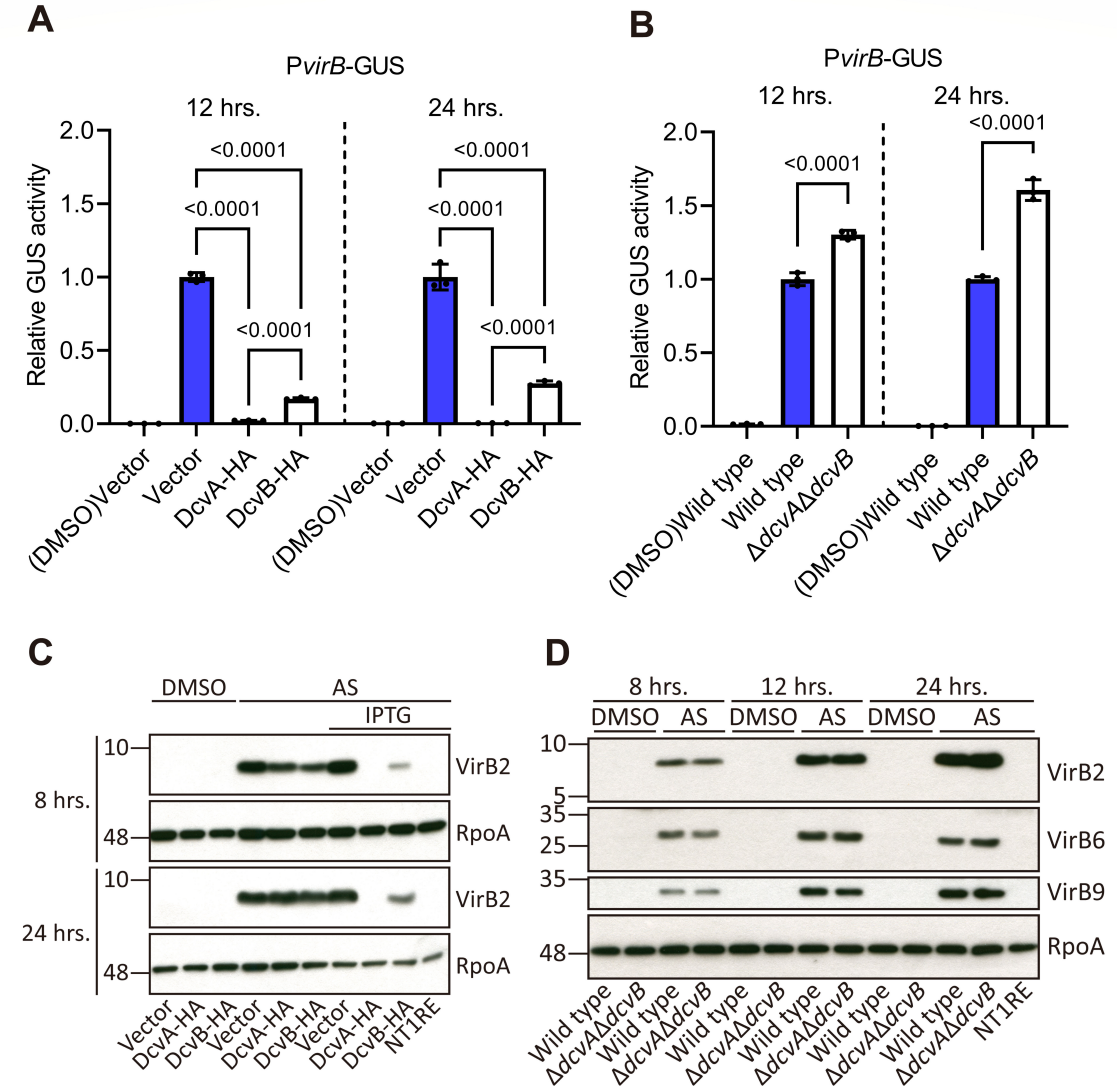
DcvA and DcvB inhibit the virulence gene expression at the transcriptional level. (A, B) Promoter activities of *virB* determined by the quantitative GUS activity with overexpression strains and double mutant carrying the reporter plasmid after AS induction. DMSO indicates the non-induced control. Relative GUS activity is calculated using the 4-MU product and normalized to the empty vector control or wild-type C58 with AS induction at each time point, set as “1.” Data are one representative result with mean ± standard deviation of three technical replicates. The number atop each bar represents the *P*-value of two-way analysis of variance with Fisher’s LSD test obtained by comparing to the vector control or wild type with AS induction. (C, D) Accumulation of virulence proteins in overexpression strains and the double mutant after AS induction. α-RpoA is included as an internal control, and the NT1RE strain without Ti plasmid is included as a negative control. Comparable results were observed in three independent experiments.

To investigate whether the inhibition of virulence by DcvA and DcvB is due to the suppression of *vir* gene induction, a *vir* promoter activity assay was performed. The *virB* or *virE* promoter fused to *gfp* (green fluorescence protein) and *gus* (β-glucuronidase) reporter genes was analyzed in *Agrobacterium* strains following acetosyringone (AS) induction. A GUS quantification assay revealed that the expression of reporter genes was significantly suppressed in strains overexpressing DcvA or DcvB (Fig. 2A; Fig. S3A). Although GFP expression could not be normalized in the DcvB overexpression strain upon IPTG induction due to cell aggregation, a significant decrease in GFP signal could be measured in strains without IPTG induction, which maintained low-level protein expression (Fig. S3B). DcvA exhibited stronger inhibition of *vir* promoter activities compared to DcvB, consistent with the observed virulence phenotypes (Fig. 1 and 2A). Conversely, the double deletion mutant Δ*dcvA*Δ*dcvB* caused larger tumors on tomato plants and enhanced the *virB* promoter activity, while no significance increase was detected in Δ*dcvA* and Δ*dcvB* single mutants (Fig. 1 and 2B; Fig. S3C through E).

We further measured the mRNA and protein levels of *vir* genes. RT-qPCR results showed that overexpression of the DGCs reduced the mRNA levels of *virB2* and *virE2*. In contrast, the double deletion mutant Δ*dcvA*Δ*dcvB* increased the mRNA level of *virB2* but had no significant effect on *virE2* (Fig. S4). Similarly, VirB protein levels decreased in strains overexpressing DGCs, while a slight increase was observed in the double mutant (Fig. 2C and D).

These results, consistent with the promoter activity assay, suggest that DcvA and DcvB inhibit *Agrobacterium* virulence by downregulating the transcription of *vir* genes.

### Overexpression of DcvB, but not DcvA, results in cell aggregation and triggers biofilm formation

Our previous study showed that overexpression of DcvB, but not DcvA, results in strong Congo Red staining, suggesting hyperaccumulation of exopolysaccharides (38). c-di-GMP levels are positively correlated with exopolysaccharide production and biofilm formation but negatively correlated with swimming motility (4–6). We next investigated the roles of DcvA and DcvB in these physiological properties. Cultures of cells overexpressing DcvB, but not DcvA, exhibited decreased planktonic density compared to the wild-type C58 strain (Fig. S5A). However, no significant differences in growth were observed for single or double deletion mutants compared to the wild-type strain (Fig. S5B). Notably, decreased planktonic cell density in the DcvB-overexpression strain correlated with cell aggregation on the surface of the test tube, unlike the uniform turbidity observed in the cells with DcvA overexpression (Fig. S5C).

**Fig 3:**
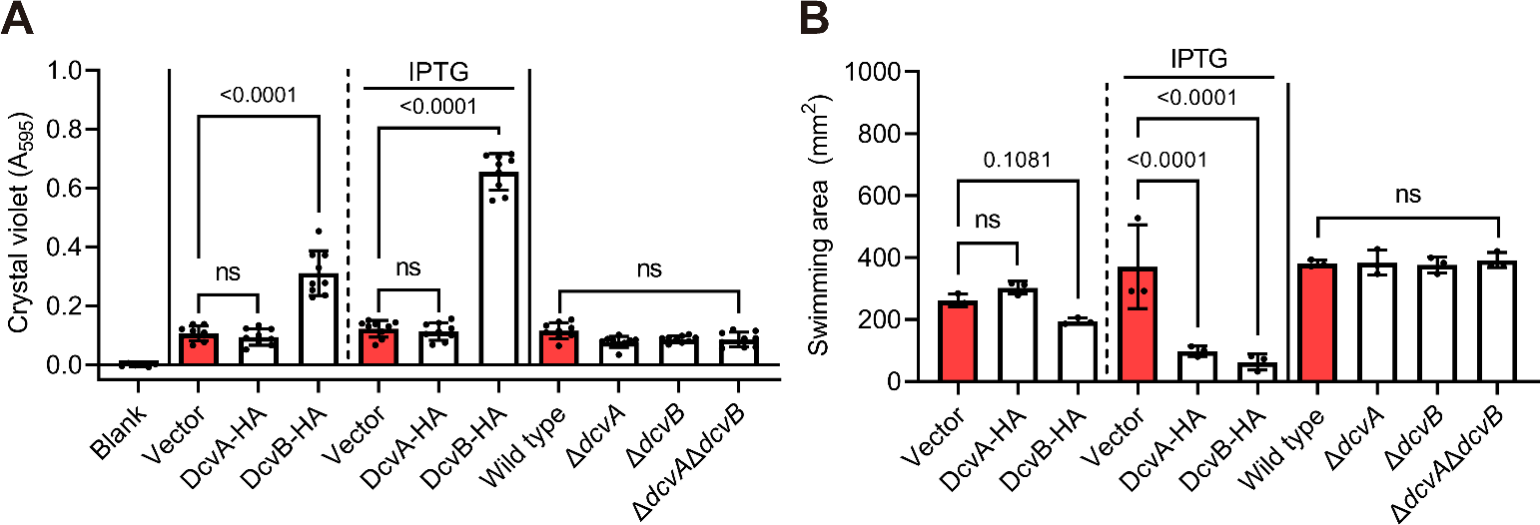
Overexpression of DcvB, not DcvA, induces biofilm formation but both reduce swimming motility. (A) Biofilm formation stained by crystal violet and quantified by absorbance value (*A*_595_) with overexpression strains and deletion mutants. Data are one representative result with mean ± standard deviation of eight technical replicates. (B) Swimming motility cultured on a 0.4% soft agar plate with overexpression strains and mutants. Data are one representative result with mean ± standard deviation of three biological replicates. Comparable results were observed in at least two independent experiments. The number atop each bar represents the *P*-value of one-way analysis of variance with Fisher’s LSD test obtained by comparing to the vector control or wild-type C58. The term “ns” denotes no significant difference (*P* > 0.01).

The observed cell aggregation in the DcvB overexpression strain suggested an increase in biofilm formation. To confirm this, we cultured strains under static conditions and stained them with crystal violet to detect biofilms. Cells overexpressing DcvB formed significantly more biofilm on the plastic surface, whereas cells with overexpression of DcvA or deletion mutants had no effect as compared to the control (Fig. 3A).

We also examined swimming motility, which often inversely correlates with biofilm formation and c-di-GMP production. Cells with overexpression of DcvA or DcvB caused reduced motility zone compared to the control (Fig. 3B). These findings suggested that DcvB plays a key role in regulating the transition from planktonic motile lifestyle to social biofilm-associated lifestyle. In contrast, DcvA has minimal effects on growth and biofilm formation but may negatively regulate motility through unknown mechanisms. Alternatively, the observed motility phenotype could result from a secondary effect or an artifact of overexpression.

Because neither single nor double mutants exhibited growth or biofilm phenotypes, we suggest that the increased virulence observed in Δ*dcvA*Δ*dcvB* is not due to the effect on these physiological changes (Fig. 1D).

### Overexpression of DcvB increases intracellular c-di-GMP via the GGEEF motif, while DcvA does not

Hyperaccumulation of c-di-GMP synthesized by DGC is known to stimulate biofilm formation (6). Given the distinct effects of DcvA and DcvB on swimming motility and biofilm formation, we examined the integrity of their catalytic GGEEF motifs through sequence alignment. Both DcvA and DcvB contain the conserved GGEEF motif and have seven predicted transmembrane domains at their N-termini (Fig. S6A and B). Structural modeling using AlphaFold3 and alignment with Chimera software revealed high structural similarity to each other in the DGC domain (Fig. S6C) (39).

Despite these similarities, only DcvB significantly impacts biofilm formation (Fig. 3). Thus, we hypothesize that DcvA does not exert DGC activity *in vivo*. Catalytically inactive variants of both DGCs (GGEEF to AAAEF) were generated to address this possibility (Fig. S6D). Unlike wild-type DcvB, cells with overexpression of DcvB^AAAEF^ did not reduce planktonic cell density (Fig. S7A). Similarly, while cells overexpressing wild-type DcvB strongly enhanced biofilm formation, the catalytic mutant completely lost this ability. Neither cells overexpressing wild-type DcvA nor DcvA^AAAEF^ affected biofilm formation (Fig. S7B).

**Fig 4:**
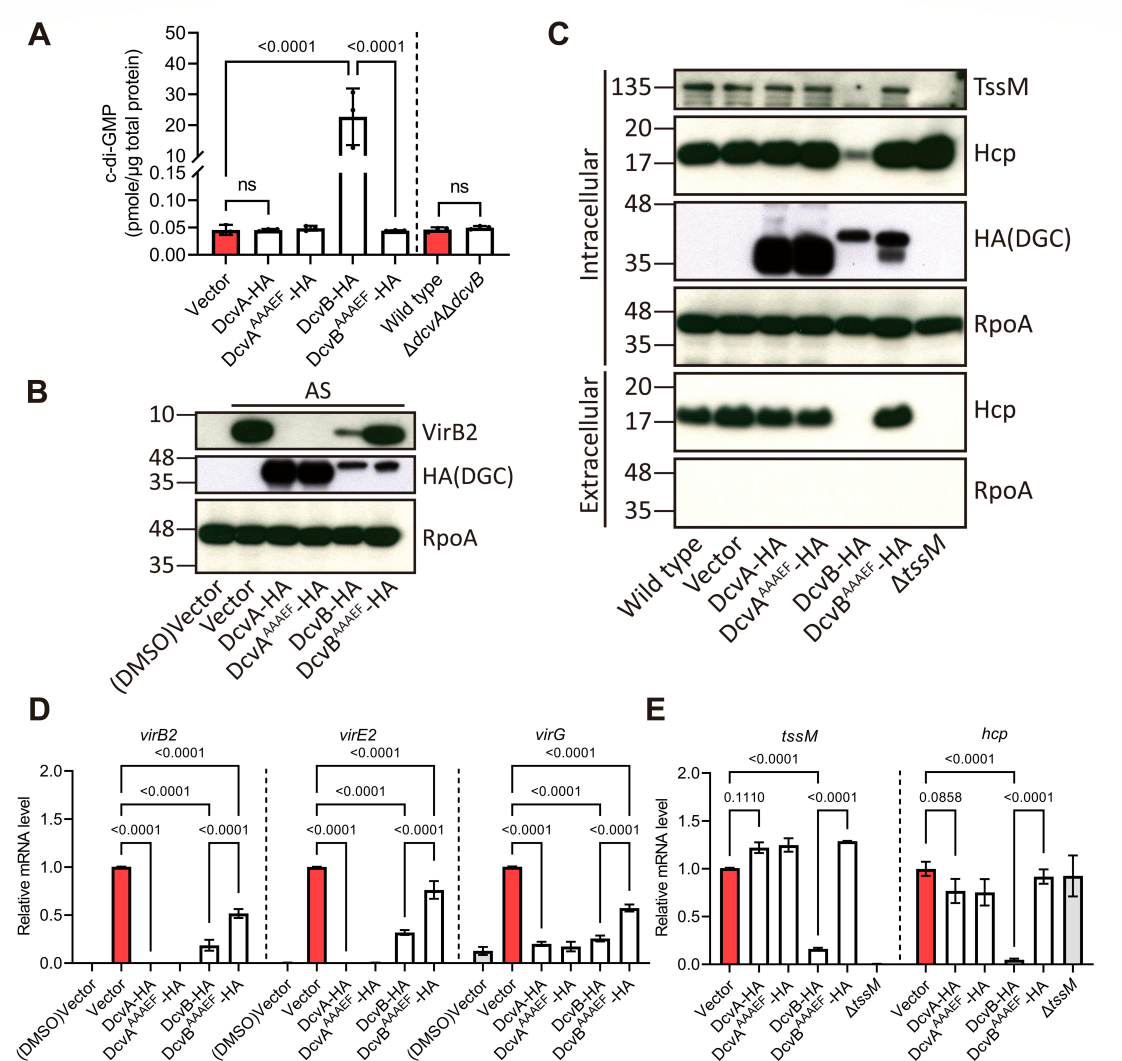
Overexpression of DcvB inhibits virulence and T6SS by catalyzing the c-di-GMP production. (A) Intracellular c-di-GMP level measured by LC–MS/MS with overexpression strains and the double mutant after the AS-induced culture. Catalytically inactive strains were generated by substituting GGEEF with AAAEF on the two DGCs. Data are one representative result with mean ± standard deviation of three biological replicates. The numbers atop each bar represent the *P*-value of one-way analysis of variance (ANOVA) with Fisher’s LSD test obtained by comparing to the vector control or wild-type C58. The term “ns” denotes no significant difference (*P* > 0.01). (B, C) Accumulation of virulence protein and T6SS components in overexpression strains after culturing in acidic minimal medium. AS was added for virulence induction, and DMSO indicates non-induced control. The Δ*tssM* mutant was included as a negative control for T6SS. α-RpoA is included as an internal control, and α-HA is included to detect the overexpression of HA-tagged DGC. Comparable results were observed in three independent experiments. (D, E) mRNA level of *vir* and T6SS genes quantified by RT-qPCR in overexpression strains after culturing in acidic minimal medium. Relative mRNA levels were calculated using the 2^−∆∆Ct^ method and normalized to the vector control with AS induction, set as “1.” Data are mean ± standard error of the mean of three independent replicates. The number above each bar represents the *P*-value of two-way ANOVA with Fisher’s LSD test obtained by comparing to the vector control with AS induction.

To confirm these observations, intracellular c-di-GMP levels were quantified using liquid chromatography coupled with tandem mass spectrometry (LC–MS/MS). Results showed that cells with overexpression of DcvB exhibited higher intracellular c-di-GMP levels, whereas those overexpressing DcvA exhibited a comparable level to the vector control (Fig. 4A). The catalytic variant DcvB^AAAEF^ loses its ability to increase c-di-GMP levels, while there is no additional effect exerted by introducing the AAAEF mutation to DcvA. Furthermore, no difference in intracellular c-di-GMP levels could be detected between wild-type C58 and the Δ*dcvA*Δ*dcvB* double mutant (Fig. 4A).

These findings demonstrate that DcvB is an active DGC, with its GGEEF motif essential for c-di-GMP synthesis. In contrast, DcvA does not exhibit detectable DGC activity *in vivo*.

### Overexpression of DcvB suppresses expression of *vir* and T6SS genes in a c-di-GMP-dependent manner, while DcvA regulates virulence independent of c-di-GMP

We next investigate the role of the GGEEF motif on DcvA and DcvB in regulating virulence and T6SS. As expected, cells with overexpression of wild-type DcvA or DcvB suppress the accumulation of VirB2. However, while VirB2 protein levels are partially rescued in the catalytic mutant of DcvB (DcvB^AAAEF^), DcvA^AAAEF^ retained the ability to inhibit VirB2 expression (Fig. 4B). Cells overexpressing DcvB also reduced intracellular protein levels of the T6SS components, TssM and Hcp, resulting in minimal secretion of hallmark protein Hcp (Fig. 4C), consistent with previous findings (38). In contrast, DcvB^AAAEF^ failed to suppress T6SS. Neither wild-type DcvA nor DcvA^AAAEF^ had any effect on T6SS protein levels or secretion (Fig. 4C).

RT-qPCR analysis also revealed that the intact GGEEF motif is important for DcvB-mediated suppression of both *vir* and T6SS gene expressions (Fig. 4D and E). Cells overexpressing DcvB^AAAEF^ partially rescued *vir* gene expression, suggesting DcvB inhibits *vir* gene expression partly via c-di-GMP-dependent mechanisms (Fig. 4D). As for DcvA, DcvA^AAAEF^ retained its ability to suppress *vir* gene expression, and, as expected, had no significant effects on T6SS gene expression, similar to wild-type DcvA (Fig. 4D and E). To further dissect the regions of DcvA required for inhibition, truncated DcvA proteins were also generated. Cells with overexpression of the N-terminal transmembrane region (DcvA) effectively suppressed Vir protein accumulation, while the cytosolic DGC domain (DcvA^DGC^) had no inhibitory effect (Fig. S8).

These findings suggest that DcvB negatively regulates virulence and T6SS via c-di-GMP synthesis, whereas DcvA inhibits virulence through its N-terminal transmembrane region in a c-di-GMP-independent manner.

### DcvA inhibits virulence downstream of VirA

VirA/VirG is the key two-component system to activate the transcription of *vir* genes, while ChvG/ChvI is involved in activating the transcription of both *virG* and T6SS genes (18, 25). Based on the evidence that both DcvA and DcvB suppress virulence induction, while only DcvB inhibits T6SS at the transcriptional level (Fig. 2 and 4), we hypothesize that DcvA targets VirA/VirG to inhibit virulence induction, while DcvB targets ChvG/ChvI to suppress both virulence and T6SS activity. To test the hypotheses, we utilized the phosphomimic response regulator capable of activating downstream gene expression, independent of their respective sensor kinase (Fig. S9).

**Fig 5:**
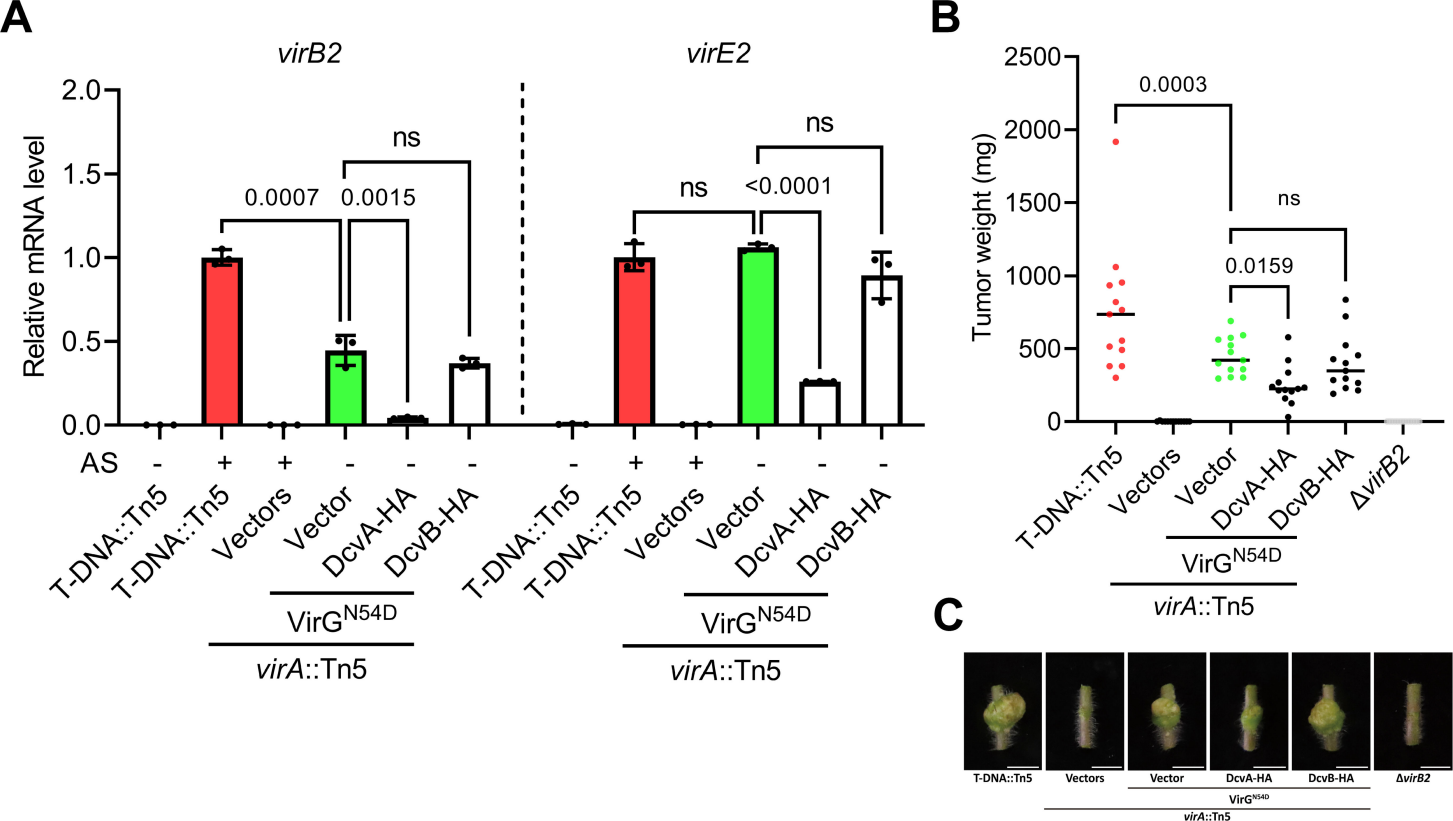
Overexpression of DcvA inhibits virulence induction and tumor formation in the phosphomimic VirG^N54D^ strain. (A) mRNA level of *vir* genes quantified by RT-qPCR in phosphomimic VirG^N54D^ strains after culturing in acidic minimal medium for 8 h. A C58-derived virulent strain, T-DNA::Tn5, is a C58-derived virulent strain with Tn5 insertion in the non-coding region of the T-DNA, which served as a positive control of AS induction for comparison to the *vir* induction level while constitutively expressing VirG^N54D^ in the *virA* mutant (*virA*::Tn5) (41). AS was added for virulence induction, and DMSO was added as non-induced control. Relative mRNA levels were calculated using the 2^−∆∆Ct^ method and normalized to T-DNA::Tn5 with AS induction in each target gene, set as “1.” Data are one representative result with mean ± standard deviation of three biological replicates. The number atop each bar represents the *P*-value of Student’s *t*-test. The term “ns” denotes no significant difference (*P* > 0.05). (B) Virulence assay measuring the tumor weight based on the tumors induced on wounded tomato stems with overexpression strains. Each dot represents the weight of each tumor (*n* = 13), and the horizontal line indicates the median. Comparable results were observed in two independent experiments. The number atop each bar represents the *P*-value of one-way analysis of variance with Fisher’s LSD test obtained by comparing to the vector control in the VirG^N54D^ strain. The term “ns” denotes no significant difference (*P* > 0.05). (C) Photographs of representative tumors. The scale bars represent 1 cm.

The N54D substitution of VirG has been demonstrated to activate the downstream *vir* gene expression in the absence of VirA or the phenolic inducer AS (40). Thus, in a *virA* mutant (*virA*::Tn5) expressing the phosphomimic VirG (VirG^N54D^), *vir* gene expression was detected at both mRNA and protein levels, albeit at lower levels compared to wild-type strains induced with AS (Fig. 5A; Fig. S10) (41). Notably, overexpression of DcvA retained the ability to suppress *vir* gene expression in the phosphomimic VirG^N54D^ strain (Fig. 5A; Fig. S10). Accordingly, the tumor assay on tomato stems also showed that the *virA*::Tn5(VirG^N54D^) strain overexpressing DcvA induced smaller tumors as compared to that of vector control. In contrast, DcvB no longer suppressed the *vir* gene expression level or tumor weight in the *virA* mutant expressing phosphomimic VirG^N54D^ (Fig. 5). These findings suggest that DcvA suppresses the expression of *vir* genes downstream of VirA, while DcvB acts upstream or independent of the VirA/VirG two-component system.

### DcvB inhibits T6SS and *virG* transcription downstream of ChvG

**Fig 6:**
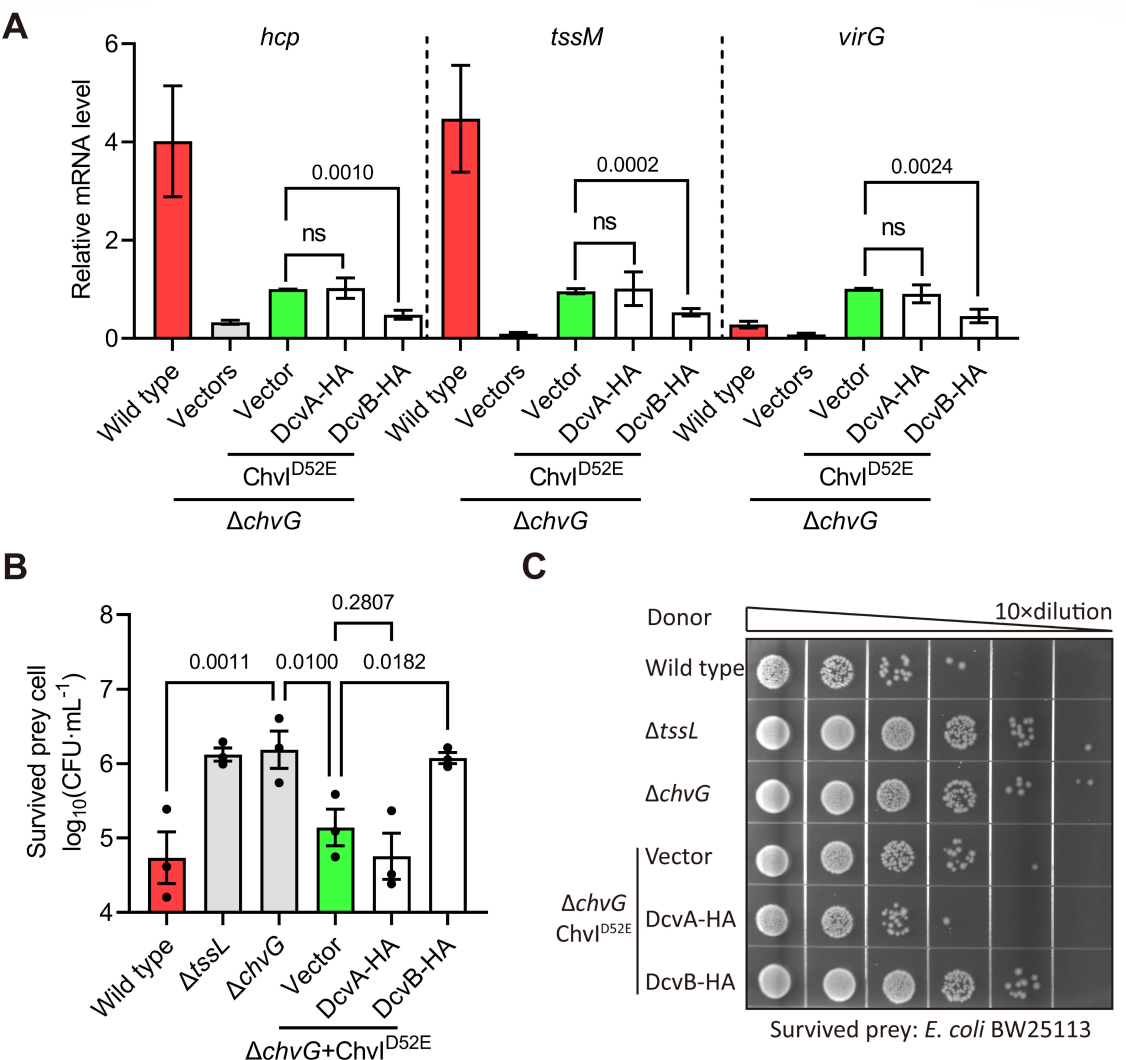
Overexpression of DcvB inhibits the expression of T6SS genes and interbacterial competition in phosphomimic ChvI^D52E^ strain. (A) mRNA levels of *virG* and T6SS genes quantified by RT-qPCR in phosphomimic ChvI^D52E^ strains after culturing in acidic minimal medium for 6 h. The wild-type C58 strain was included as a positive control for virulence induction, while Δ*chvG* was included as a negative control. Relative mRNA levels were calculated using the 2^−∆∆Ct^ method and normalized to Δ*chvG* carrying pChvI^D52E^ to constitutively express ChvI^D52E^, set as “1.” Data are presented as mean ± standard deviation of six biological replicates, with each biological replicate being the average of three technical replicates. The number above each bar represents the *P*-value of Student’s *t*-test obtained by comparing to the Δ*chvG-*expressing ChvI^D52E^ strain. The term “ns” denotes no significant difference (*P* > 0.01). (B, C) Interbacterial competition quantified by survived prey cells after co-incubating ChvI^D52E^ strains and *E. coli* BW25113 on AK medium for 16 h. Δ*tssL* was included as a negative control for T6SS. Data are presented as mean ± standard error of the mean of three independent replicates. The number atop each bar represents the *P*-value of one-way analysis of variance with Fisher’s LSD test obtained by comparing to the vector control in the ChvI^D52E^ strain.

We next tested the hypothesis that DcvB downregulates both virulence and T6SS activity via ChvG/ChvI, the pH global regulator (25, 33–35). To test this, we employed an approach similar to that used for VirA/VirG (Fig. S9). The ChvI^D52E^ mutant renders it constitutively active and enhances the T6SS gene expression, even under neutral pH conditions (25). The phosphomimic ChvI^D52E^ was constitutively expressed in a Δ*chvG* mutant to eliminate the effect of ChvG activation under acidic conditions. RT-qPCR results showed that ChvI^D52E^ in the Δ*chvG* mutant increases T6SS gene expression, though to a lesser extent than in the wild-type C58. In the Δ*chvG*(ChvI^D52E^) phosphomimic strain, overexpression of DcvB significantly suppressed T6SS gene expression, whereas DcvA had no significant effect (Fig. 6A). These findings indicate that DcvB inhibits T6SS activation by targeting transcription downstream of ChvI. In addition, since phosphorylated ChvI upregulates *virG* transcription as well (33–35), we measured *virG* gene expression in Δ*chvG*(ChvI^D52E^). Results showed that ChvI^D52E^ increased *virG* expression in the *ΔchvG* mutant, but overexpression of DcvB, not DcvA, suppressed this effect (Fig. 6A). The findings suggest that DcvB inhibits expressions of *virG* and T6SS genes at step(s) downstream of ChvG.

To assess the functional impact of this regulation, we evaluated interbacterial competition by co-incubating Δ*chvG*(ChvI^D52E^) strains with *E. coli* prey cells. Results showed a higher survival rate of *E. coli* prey cells when co-cultured with DcvB overexpression strain as compared to the vector-only control (Fig. 6B and C). In contrast, DcvA overexpression in Δ*chvG*(ChvI^D52E^) does not significantly affect the survival rate of *E. coli*. These findings reveal that DcvB suppresses *vir* and T6SS gene expression by targeting steps downstream of ChvG; and as expected, DcvA does not appear to suppress T6SS.

## DISCUSSION

**Fig 7:**
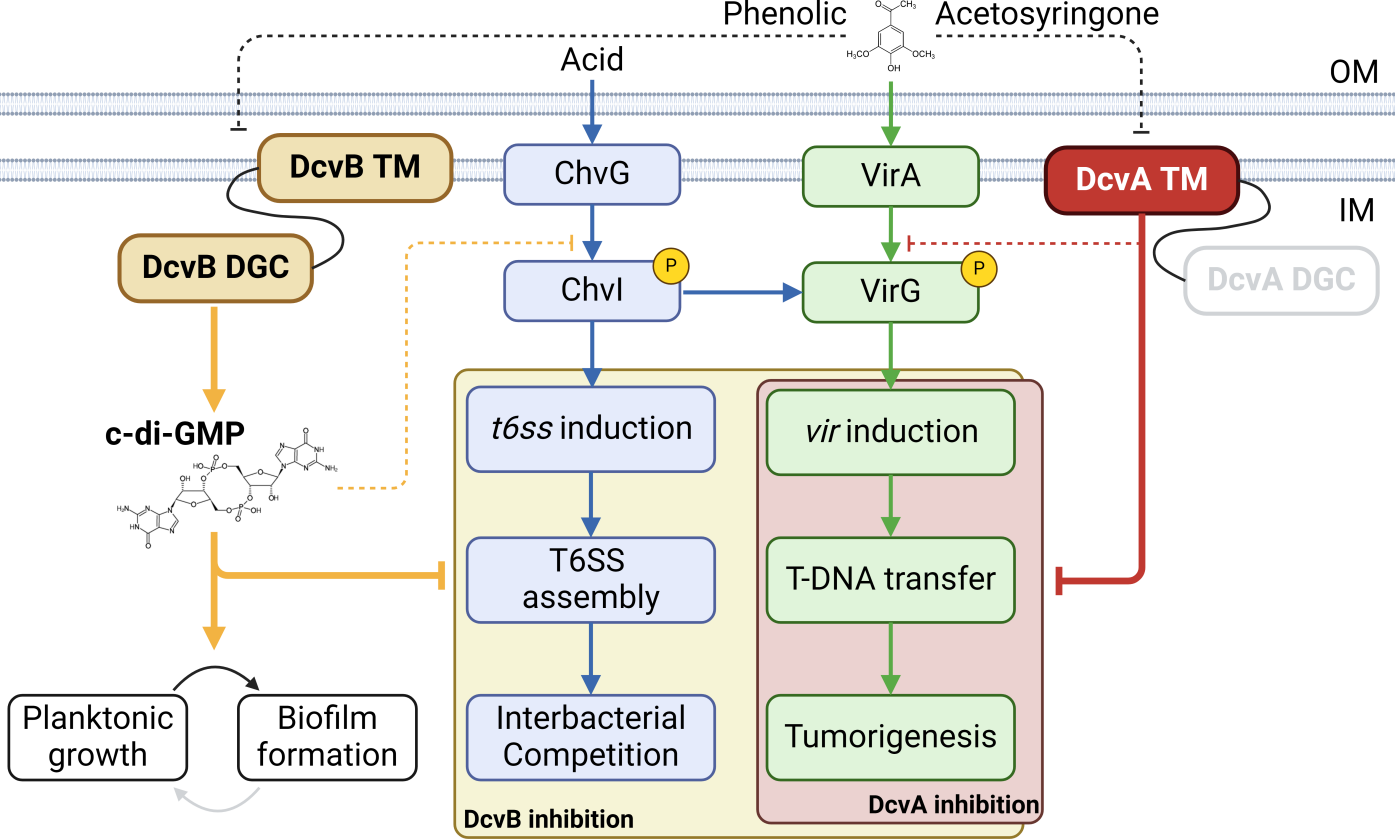
Model of DcvA and DcvB regulating virulence and T6SS in *Agrobacterium* C58. DcvA targets downstream of VirA independent of c-di-GMP, resulting in the suppression of downstream transcription of *vir* genes. On the other hand, DcvB catalyzes c-di-GMP production, which not only triggers biofilm formation but also leads to the suppression of the downstream transcription of both *vir* and T6SS genes. The solid line shows the regulation demonstrated in this study, while the dashed line indicates putative or undefined regulation. “OM” indicates outer membrane, and “IM” indicates inner membrane. The figure was created using Biorender.com.

In this study, we discovered that two putative transmembrane diguanylate cyclases (TM-DGCs), DcvA and DcvB, act as negative regulators of virulence in *Agrobacterium*, operating through distinct mechanisms. DcvB elevates the intracellular c-di-GMP levels, promoting biofilm formation while simultaneously reducing planktonic cell growth, motility, virulence, and T6SS in a c-di-GMP-dependent manner. The GGGEF domain substitution analysis further provided evidence that DcvB possesses functional diguanylate cyclase activity. Conversely, DcvA does not increase cellular c-di-GMP level but inhibits virulence likely by binding locally with an unknown partner. In conclusion, DcvB functions as a global regulator affecting multiple biological processes, while DcvA specifically regulates virulence independent of c-di-GMP (Fig. 7).

Bacteria employ both global and local modes of c-di-GMP signaling. Master DGCs and PDEs control the intracellular c-di-GMP level to activate effectors, while some DGCs interact directly with specific effectors (42, 43). Elevated c-di-GMP levels activate cellulose synthase, promoting cellulose biosynthesis and biofilm formation (44). In *Agrobacterium*, unipolar polysaccharide and cellulose biosynthesis are involved in bacterial attachment through stimulation by c-di-GMP (45). These findings suggest that elevated levels of c-di-GMP in bacterial cells alter bacterial physiology for initial adhesion. In this study, DcvB functions as a global regulator by increasing c-di-GMP levels to regulate multiple biological processes, including biofilm formation and motility (Fig. 4). DcvB-enhanced c-di-GMP also suppresses virulence and T6SS-dependent interbacterial competition likely by targeting the components downstream of ChvG, such as ChvI (Fig. 6). Further research is required to identify the c-di-GMP binding regulators downstream of ChvG that are involved in activating the transcription of both *vir* and T6SS genes.

Not all annotated DGCs contribute to c-di-GMP production. For instance, DgcC (Atu2179) in *Agrobacterium* C58 does not modulate the intracellular c-di-GMP level (37) but inhibits T6SS-mediated interbacterial competition (38). Amino acid alignment revealed low sequence similarity between DvcA and DvcB in their N-terminal transmembrane region despite sharing a conserved C-terminal DGC domain (Fig. S6C and S11). DcvA does not contribute to the global c-di-GMP pool production, and its transmembrane region alone is sufficient to suppress virulence (Fig. S8). This suggests that the DGC domain of DcvA is either degenerate or catalytically inactive under the conditions tested. Current evidence indicates that DcvA suppresses the transcription of *vir* genes, independent of c-di-GMP by interfering with regulatory pathways downstream of VirA (Fig. 5). Potential targets include VirG or other unidentified factors involved in activating virulence. A recent study demonstrated that the cytosol-exposed region of Dma1 (dual membrane-spanning anti-sigma factor) directly interacts with a cytosolic sigma factor, affecting vesiculation in *Bacteroides thetaiotaomicron* (46). We hypothesize that the cytosolic, membrane-exposed region of DcvA may directly interact with cytosolic VirG, preventing it from activating *vir* gene expression.

Environmental signals influence DGC and PDE activation (47). Sensory domains typically located in the N-terminal region detect stimuli, such as light, temperature, quorum-sensing, and other small molecules. However, no predictable sensory domain is present in the N-terminal regions of both DcvA and DcvB, suggesting that their regulatory function may depend on the expression levels. RNA-Seq analyses have revealed that several *dgc*/*pde* genes, including *dcvA* and *dcvB*, are downregulated in *Agrobacterium* C58 in response to AS or plant signals (48, 49). This observation suggests a plausible model that *Agrobacterium* downregulates these genes during plant colonization to enhance T6SS and *vir* gene expressions, facilitating niche competition and infection. Future transcriptome analysis of the Δ*dcvA*Δ*dcvB* double mutant and overexpression strains *in planta* will be crucial to test this hypothesis.

Gene redundancy complicates functional studies of individual DGC genes, as single mutants often exhibit no detectable phenotypes. In our study, single deletions of *dcvA* or *dcvB* showed no phenotypes, indicating functional compensation among DGCs. However, the Δ*dcvA*Δ*dcvB* double mutant exhibited increased *vir* gene expression, underscoring the biological significance of these two DGCs in virulence suppression (Fig. 2B). We observed that the Δ*dcvA*Δ*dcvB* double mutant increases tumor formation (Fig. 1D) while showing no significant effect on transient transformation efficiency in *Arabidopsis* seedlings (Fig. S2). This discrepancy could be due to the difference in the plant hosts or methods (transient versus stable transformation) used to determine the phenotypes. Future work to evaluate the *Agrobacterium-*mediated transient and stable transformation efficiencies on tomato plants of the Δ*dcvA*Δ*dcvB* double mutant or generating the mutants with deletion of multiple *dcv* genes in commonly used disarmed strains may hold promises for biotechnology application to plant transformation.

The double mutant displayed no changes in the intracellular c-di-GMP level compared to the wild type (Fig. 4A). These observations suggest c-di-GMP production by DcvB can be compensated for with other DGCs, and the elevated *vir* gene expression in Δ*dcvA*Δ*dcvB* is independent of c-di-GMP. Previous studies also showed that overexpression of PleD strongly forms cell aggregation and suppresses virulence, while single deletion of *pleD* in *Agrobacterium* C58 had no effect on biofilm formation, motility, unipolar polysaccharide production, and tumor formation (36, 37). Similarly, overexpression of *dgcA*, *dgcB*, or *dgcC* in *Agrobacterium* C58 enhanced the overall c-di-GMP and cellulose production in the cell, but no phenotypes were observed in single mutants (37). These findings highlight functional redundancy within the c-di-GMP signaling network in *Agrobacterium*. Future work combining overexpression and single and multiple deletions of *dgc* genes shall shed light on understanding their common and specific regulatory roles.

## MATERIALS AND METHODS

### Bacterial strains and growth conditions

The strains and plasmids used in this study are detailed in Tables S1 and S2. *Agrobacterium* strains were cultured in 523 medium (pH 7.0) at 28°C (50), and *E. coli* strains were cultured in Luria Bertani (LB) medium at 37°C, unless otherwise specified. Virulence genes in *Agrobacterium* were induced by culturing the bacterial cell in acidic AB-MES medium (pH 5.5) with 200 µM AS at 28°C (51). Antibiotic selection was performed using medium supplemented with 100 µg/mL spectinomycin (Sp^R^), 50 µg/mL gentamycin (Gm^R^), 50 µg/mL kanamycin (Km^R^), and 100 µg/mL ampicillin (Ap^R^).

### Construction of plasmids and mutants

Plasmids pTrc200 and pRLop are used for protein expression, and primers utilized in this study are detailed in Table S3. The gene of interest was amplified via polymerase chain reaction (PCR), followed by restriction enzyme digestion. The digested fragment was ligated downstream of the promoter in the vector plasmid, and the construct was verified by Sanger sequencing. Gene expression was induced by 1 mM isopropyl β-d-1-thiogalactopyranoside (IPTG) in *Agrobacterium* carrying the expression plasmid, unless otherwise specified. For the generation of in-frame deletion, 500 bp sequences upstream and downstream of the gene were fused and ligated into the suicide vector pJQ200sk. Following electroporation into *Agrobacterium* cell, a two-step homologous recombination was carried out accordingly to obtain the mutants (52), which were identified by PCR, followed by DNA sequencing.

### Immunoblotting

The protein sample used for loading was derived from equal amounts of cells by normalization to O.D._600_ 10. Tris–glycine sodium dodecyl sulfate–polyacrylamide gel electrophoresis (SDS-PAGE; 12 or 15%) was applied for protein separation. After electrophoresis and PVDF transfer, the primary antibody was added and incubated at 4°C with shaking overnight (α-TssM, 1:1,500; α-VirB6/VirB9, 1:2,000; α-Hcp, 1:2,500; α-VirB2, 1:3,000; α-HA/EF-Tu/RpoA/VirE2, 1:5,000). The membrane was washed with TBST and blocked with skimmed milk again for secondary antibody recognition (α-rabbit/mouse, 1:20,000). The washed membrane was then incubated with western chemiluminescent HRP substrate and exposed to the high-performance chemiluminescence film to be developed by the X-ray film processor.

### Transient transformation assay

To assess the transient transformation efficiency, this study employed *Agrobacterium*-mediated enhanced seedling transformation as outlined in prior research (53). *Arabidopsis thaliana* Col-0 seedlings were germinated using half-strength MS medium (0.217% Murashige and Skoog salt mixture, 0.5% sucrose, pH 5.7, with KOH) at 22°C under a 16/8 h day/night cycle (53). Strains harboring the reporter plasmid pBISN1 with a *gus*-intron were cultured in AB-MES (pH 5.5) containing 200 µM AS for 16 h for virulence induction and resuspended in infection medium (comprising 50% AB-MES, 50% half MS, 200 µM AS, and 1 mM IPTG, pH 5.5) to O.D._600_ 0.02. The bacterial suspension was incubated with 4 day-old seedlings in each well of six-well plate for 2 days. The seedlings were subjected to GUS stains by X-Gluc and GUS activity assay by 4-methylumbelliferyl-β-D-glucuronide (MUG). The fluorescence intensity was measured in a dark 96-well plate under emission/excitation: 365/455 nm using a Synergy H1 hybrid multi-mode microplate reader. Fluorescence values were analyzed by Gen5 software and normalized by both total protein concentration and standard 4-methylumbelliferone (4-MU; the final product of MUG).

### Tumor assay

For the tumor assay, tomato seeds cultivar Known-You 301 (Known-You Seed Co., Ltd., Taiwan) were planted in 3 in. pots filled with a mixture of Jiffy premium fine peat moss substrate, perlite, and vermiculite (4:1:1) in the greenhouse. A sterilized needle was used to create a wound on the stem between two cotyledons of 4 week-old plants, followed by inoculation of O.D._600_ 1.0 bacterial suspension in 0.9% NaCl. The tumor was excised using a scalpel for biomass measurement at 4 weeks post-inoculation.

### Promoter activity assay

Transcriptional fusion of *virB* or *virE* fused with fluorescence reporter genes *gus* and *gfp* (green fluorescent protein) on pRU1156 reporter plasmid (54) was used for promoter activity assay. Strains carrying reporter plasmid were cultured in AB-MES (pH 5.5) containing 200 µM AS for virulence induction. Bacterial cells were collected at different time points for both GUS and GFP quantifications. For the GUS assay, cells were resuspended in PBS buffer (137 mM NaCl, 10 mM Na_2_HPO_4_, 2.7 mM KCl, 1.8 mM KH_2_PO_4_) containing 0.001% SDS and 10% chloroform for cell lysis. After 10,000 × *g* centrifugation, the supernatant was mixed with PBS buffer containing 0.1 mM MUG and transferred to the black 96-well plate immediately to measure the fluorescence intensity for quantification as described above. For GFP quantification, cells were resuspended in 0.9% NaCl and adjusted to O.D._600_ 0.2. Next, 200 µL suspension was added into 96-well plate, and the fluorescence intensity was measured under emission/excitation: 395/509 nm with synergy H1 hybrid multi-mode microplate reader. The relative fluorescent unit was quantified by plate reader without background value.

### Reverse transcription and quantitative PCR (RT-qPCR)

To determine the mRNA level of genes, *Agrobacterium* strains were cultured in AB-MES (pH 5.5) containing 200 µM AS for virulence gene induction. Total RNA was extracted by commercial kit (ARROWTEC, Taiwan), and the final product was dissolved with DNase/RNase-free distilled water. An equal amount of DNase-treated RNA was used to generate complementary DNA (cDNA) by reverse transcriptase (GRISP, Portugal). SYBR green PCR reagent (Thermo Fisher Scientific, USA) was applied to determine the mRNA level by QuantStudio 12K Flex Real-time PCR System (Thermo Fisher Scientific, USA). Primer pairs used for qPCR are listed in Table S1. The relative mRNA expression level was calculated based on the Ct value and normalized by 16s rRNA gene as an internal control.

### Planktonic cell growth

Bacterial strains were cultured in the AB-MES (pH 5.5) containing 200 µM AS with adjusted O.D._600_ 0.2. The suspension was transferred to the test tube and incubated at 28°C with 230 rpm shaking. The optical density was measured at 600 nm wavelength every 1 h after vortexing.

### Biofilm assay

For quantification of the biofilm formation, bacterial strains were prepared in the AB-MES (pH 5.5) containing 200 µM AS with adjusted O.D._600_ 0.2. Next, 3 mL of bacterial suspension was transferred into each well of 12-well plate and cultured at 28°C for 48 h without shaking. The liquid culture was carefully discarded, and the plate was washed by gently rinsing in water. Then, 3 mL of 0.1% (wt/vol) crystal violet was added into each well and stained the cells in biofilm for 5 min. Dye was discarded, and the well plate was washed again by water. The adherent dye was solubilized with 95% ethanol for 5 min, and the solution was transferred into the 96-well plate to measure the absorbance value at 595 nm wavelength by Synergy H1 hybrid multi-mode microplate reader.

### Swimming motility assay

Bacterial strains were prepared in sterilized distilled water with adjusted O.D._600_ 1.0 Bacterial suspension was injected into the center of AB-MES (pH 5.5) plate containing 0.4% (wt/vol) agar and 200 µM AS. The plate was incubated at 28°C for 72 h, and the image was taken by a Scan 500 automatic colony counter. To measure the swimming area of the swimming rings, ImageJ software was used to calculate the size of the colony.

### Sequence comparison and structure prediction *in silico*

The sequences of each TM-DGC on the genome of *Agrobacterium* C58 (GCA_000092025.1) were obtained from GenBank. The amino acid sequences of TM-DGCs were aligned using multiple sequence comparison by log-expectation with MEGA 11 software. Functional domains and specific sites were predicted using Simple Modular Architecture Research Tool open resource (55) and conserved domain database search provided by the National Center for Biotechnology Information. Secondary structures and transmembrane regions were predicted using Phyre2 (56). Protein structures were predicted using AlphaFold 3 (39), and the exported structures were aligned with the matchmaker function in ChimeraX software. The root–mean–square deviation was calculated, with a lower value (usually <2 Å) indicating higher confidence in structural similarities.

### Quantification of intracellular c-di-GMP

Quantification of intracellular c-di-GMP level was adapted based on reference 57. Bacterial strains were prepared in the AB-MES (pH 5.5) containing 200 µM AS with adjusted O.D._600_ 0.2. Next, 5 mL of bacterial suspension was transferred to the test tube and incubated at 28°C for 8 h with 230 rpm shaking. An equal amount of bacterial cells was collected and resuspended in distilled water with adjusted O.D._600_ 10. Then, 2-chloro-AMP (Sigma Aldrich, USA) was added into each sample to final 0.1 µM as a technical control, and perchloric acid was added to final 0.6 M for cell lysis. After a 30 min incubation on ice, potassium bicarbonate was added to a final 0.5 M for neutralization. The supernatant was obtained by centrifuging at 20,000 × *g* for 10 min at 4°C twice and subjected to LC–MS/MS analysis. To calculate the c-di-GMP concentration, synthesized c-di-GMP (Sigma Aldrich, USA) with different concentrations was included to generate a standard curve for calculation.

### Isolation of T6SS secretory proteins

The secretion assay for detecting T6SS secretory proteins was described previously (58). Strains were cultured in AB-MES (pH 5.5) containing 1 mM IPTG starting from O.D._600_ 0.2 and incubated at 25°C for 6 h with shaking. After centrifugation with 10,000 ×*g* at 4°C for 10 min, the pellet was collected and resuspended to O.D._600_ 10 in TE buffer serving as intracellular fraction, and the supernatant was then filtered through a 0.22 µm membrane to remove any remaining cells. To disrupt protein interactions, 0.0003% sodium deoxycholate was added and incubated on ice for 10 min. Next, 15% trichloroacetic acid was added to precipitate the secretory proteins from the culture medium by incubating at 4°C overnight. Precipitated proteins were then collected via centrifugation at 10,000 ×*g* and resuspended in 1 M Tris with original pH to dissolve for immunoblotting analysis.

### Interbacterial competition assay *in vitro*

To determine interbacterial competition, bacterial cultures of donor cell *Agrobacterium* strains and prey cell *E. coli* BW25113 were prepared by adding overnight cultures into fresh medium containing 1 mM IPTG for 3 h induction. Mid-log phase bacterial cells were spun down and resuspended in 0.9% NaCl containing 1 mM IPTG with an adjusted O.D._600_ 1.0. *E. coli* prey cells were diluted 30-fold and mixed with O.D._600_ 1.0 *Agrobacterium* donor cells at a 1:1 ratio. The mixture was spotted as two 10 µL spots onto a neutral AK medium (AB-MES medium without sugar and ions) (59) containing 1 mM IPTG and 2% agar. After 16 h of incubation at 25°C, the colonies were collected, resuspended in 0.9% NaCl, and serially diluted to recover surviving prey cells by spotting onto LB medium containing antibiotics.

## SUPPLEMENTAL MATERIAL

Supplemental figures and tables mbio.00039-25-s0001.pdf Figures S1 to S11 and Tables S1 to S3.

ASM does not own the copyrights to Supplemental Material that may be linked to, or accessed through, an article. The authors have granted ASM a non-exclusive, world-wide license to publish the Supplemental Material files. Please contact the corresponding author directly for reuse.
