# Supplementary material for: Distinct strategies of diguanylate cyclase domain proteins on inhibition of virulence and interbacterial competition by agrobacteria
Source: mBio. 2025 Apr 17;16(5):e00039-25. doi: 10.1128/mbio.00039-25 (PMC12077199; doi:10.1128/mbio.00039-25)
Supplement: Supplemental figures and tables — Figures S1 to S11 and Tables S1 to S3. [file mbio.00039-25-s0001.pdf]

Supplementary Materials

Distinct strategies of diguanylate cyclase domain proteins on inhibition of virulence and interbacterial competition by agrobacteria

Xuan Lai[a,b,c], Manda Yu[a,d], Chiu-Ping Cheng[b], Erh-Min Lai[a,c*]

[a] Institute of Plant and Microbial Biology, Academia Sinica, Taiwan.
[b] Institute of Plant Biology, National Taiwan University, Taiwan.
[c] Institute of Plant Pathology and Microbiology, National Taiwan University, Taiwan.

Running title: DGC domain proteins regulating virulence and interbacterial competition

*Address correspondence to Erh-Min Lai, emlai@gate.sinica.edu.tw.
Institute of Plant and Microbial Biology, Academia Sinica, 128 Sec. 2, Academia Rd, Nangang, Taipei 115201 Taiwan.

[d] Present address: Department of Microbiology, ADA Forsyth Institute, Cambridge, MA, United States

Fig. S1

Accumulation of HA-tagged DGCs in overexpression strains.

Accumulation of HA-tagged DGCs in overexpression strains after AS induction for eight hours. 1 mM IPTG was added to drive the overproduction of DcvA and DcvB expressed from pTrc200 plasmid. α-EF-Tu is included as an internal control. Comparable results were observed in three independent experiments.

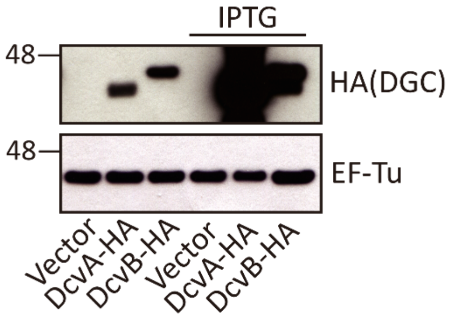

Fig. S2

Double deletion mutant Δ*dcvA*Δ*dcvB* has no effects on transient transformation efficiency in *Arabidopsis* seedlings.

Transient transformation efficiency determined by quantitative GUS activity with wild-type C58 and double mutant Δ*dcvA*Δ*dcvB* carrying the GUS-intron reporter plasmid pBISN1. The Δ*virB2* mutant is a nonpathogenic mutant. Each dot represents a biological replicate, with each replicate consisting of ten infected seedlings. Data are one representative result with mean ± SD of three biological replicates. Comparable results were observed in two independent experiments. The term "ns" indicates no significant difference (P>0.1, one-way ANOVA with Tukey HSD test).

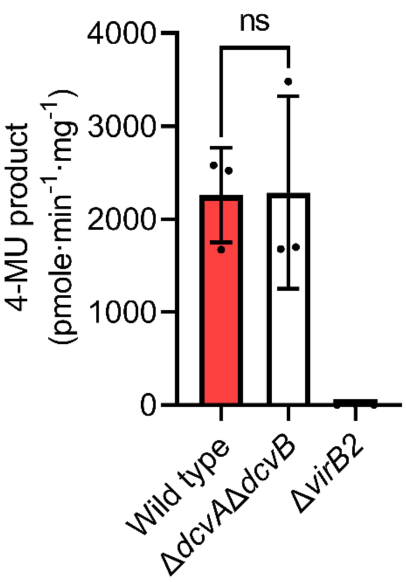

Fig. S3

DcvA and DcvB inhibit virulence gene expression at the transcriptional level.

Promoter activities of *virB* and *virE* determined by quantitative GUS activity and GFP signal with (A-B) overexpression strains and (C-E) the mutants. These strains carrying the reporter plasmid were cultured in AS induction medium for 8, 12, and 24 hours. DMSO indicates the non-induced control. Panel B shows the strains without IPTG induction for lower-level expression to address the inaccurate detection of DcvB while overproduction, which is caused by cell aggregation. Data are one representative result with mean ± SD of three technical replicates. Comparable results were observed in three independent experiments. The number atop each bar represents the *P*-value of two-way ANOVA with Fisher's LSD test obtained by comparing to the vector control or wild-type C58 with AS induction. The term "ns" denotes no significant difference (P>0.01).

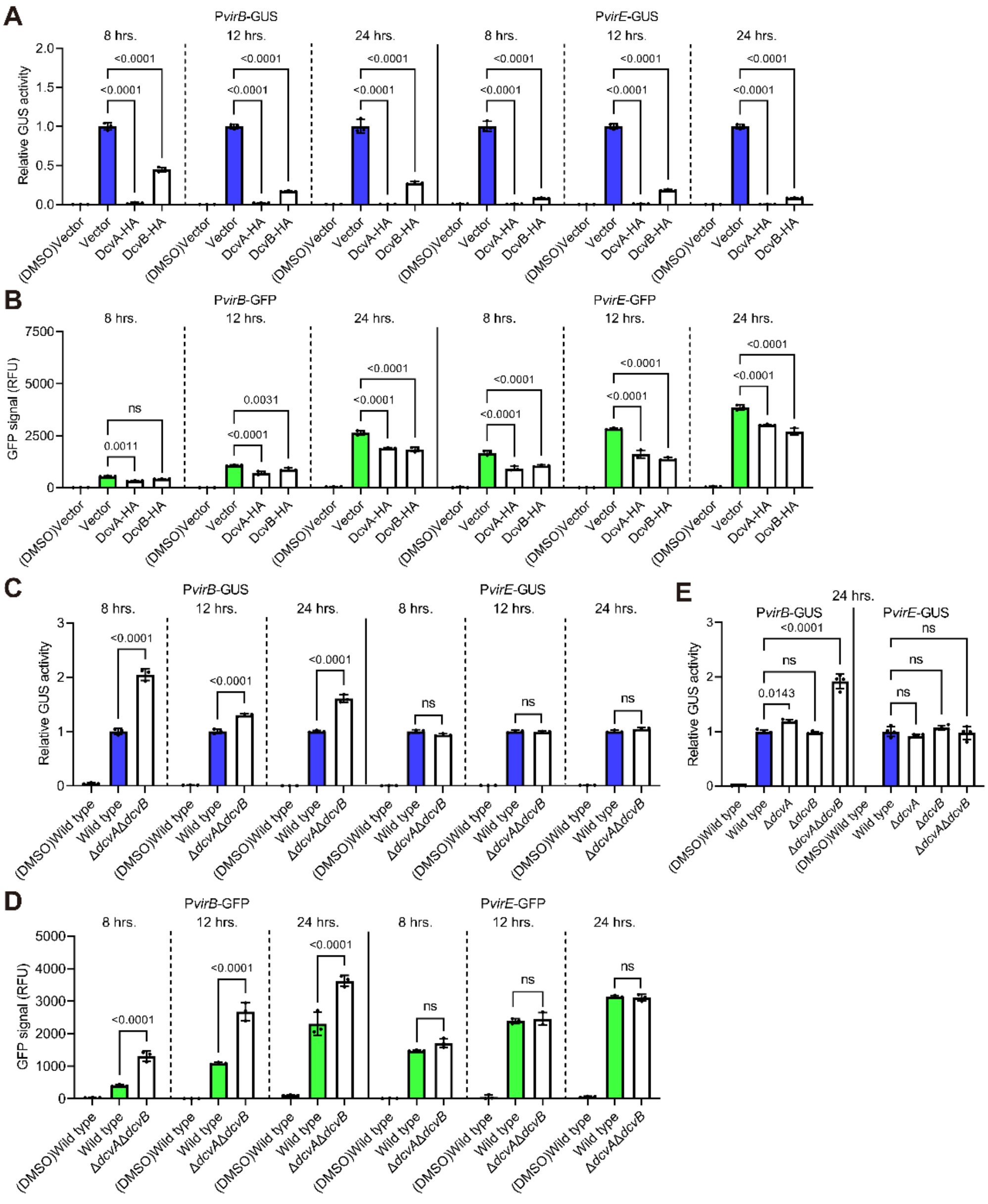

Fig. S4

DcvA and DcvB reduce mRNA level of virulence gene.

The mRNA level of *virB* and *virE* quantified by RT-qPCR in (A) overexpression strains and (B) double mutant after AS induction for eight hours. DMSO indicates the non-induced control. Relative mRNA levels were calculated using the $2^{-\Delta\Delta Ct}$ method and normalized to the vector control or wild-type C58 with AS induction, set as "1". Data are one representative result with mean ± SD of three technical replicates. Comparable results were observed in three independent experiments. The number atop each bar represents the *P*-value of Student's t-test obtained by comparing to the vector control or wild type with AS induction.

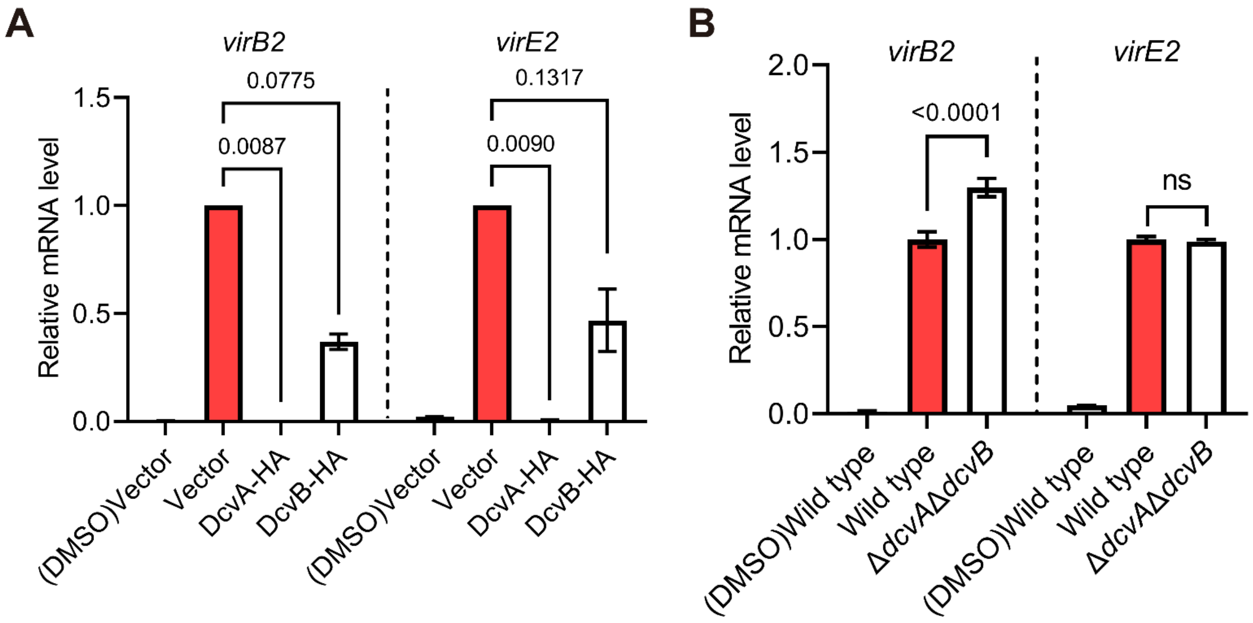

Fig. S5

Overexpression of DcvB reduces planktonic cell density and induces cell aggregation.

The planktonic cell density measured by optical density (O.D.$_{600}$) with (A) overexpression strains and (B) mutants while culturing in AS induction medium for 24 hours. In panel A, the solid line indicates the presence of IPTG induction, and the dashed line represents the absence of IPTG for low level expression. Data are one representative result with mean ± SD of three biological replicates. Comparable results were observed in three independent experiments. (C) The photo shows culture tubes during the cultivation.

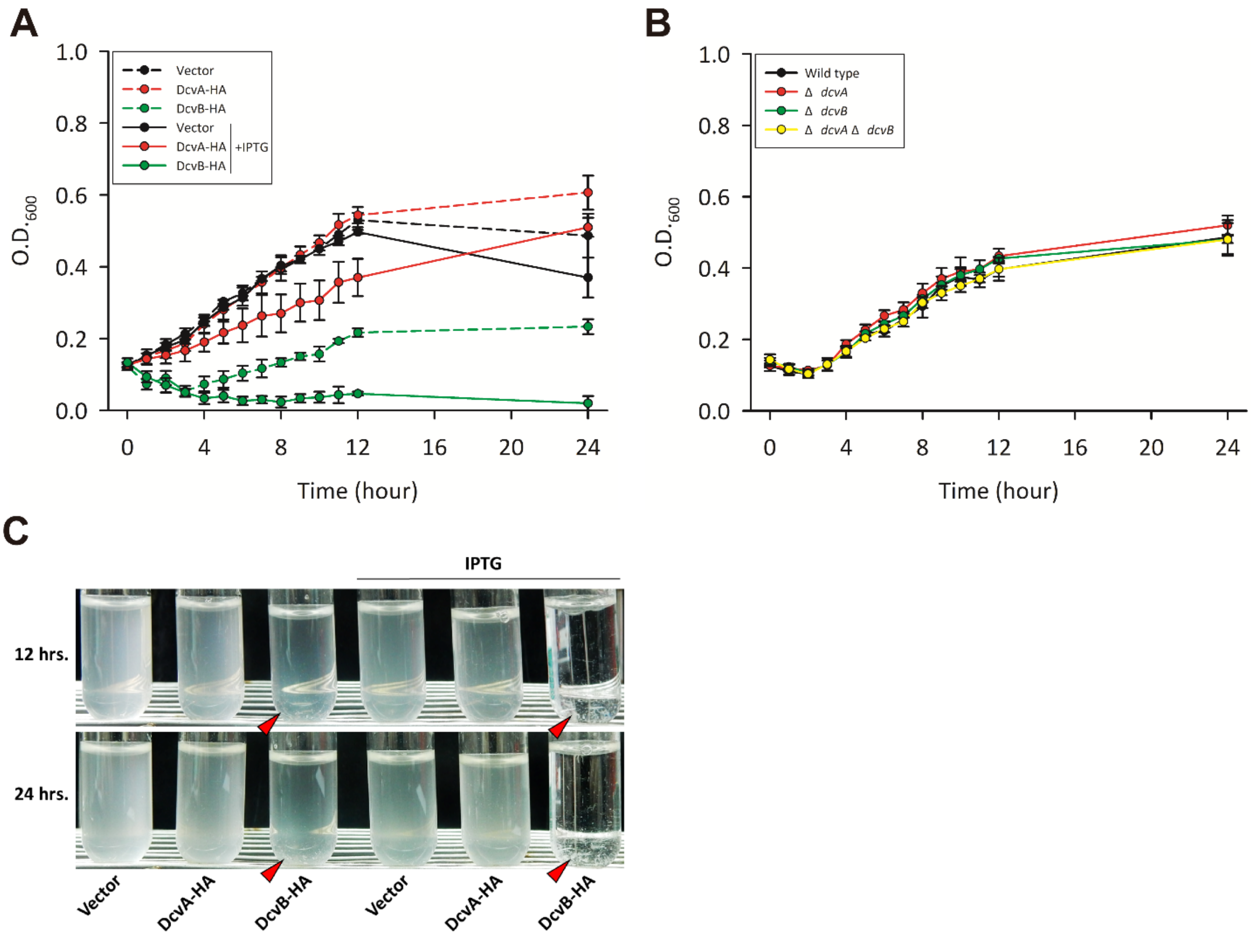

Fig. S6

DcvA and DcvB contain a conserved DGC domain.

(A) The alignment of amino acid sequences in the transmembrane-DGC proteins. The number at each site's corner indicates its amino acid position of the full-length protein. The sequences of PleD, which produces c-di-GMP and triggers biofilm formation, and other TM-DGCs including DgcA, DgcB, DgcC, Atu1060, Atu2091, Atu2691 are included as references (1). The cyan color represents the core GGEEF catalytic motif, and the yellow color indicates consensus amino acid sequences. (B) The feature prediction by SMART (2) with labeled amino acid positions of DcvA and DcvB sequences. (C) The alignment of the predicted protein structure of the DGC domain of DcvA (red) and DcvB (cyan) as determined by AlphaFold 3. The RMSD between 122 pruned atom pairs is 0.787 angstroms. (D) The alignment of the predicted protein structure of wild-type DcvB (cyan) and catalytically inactive DcvB^{AAAEF} (purple) as determined by AlphaFold 3. The RMSD between 362 pruned atom pairs is 0.749 angstroms.

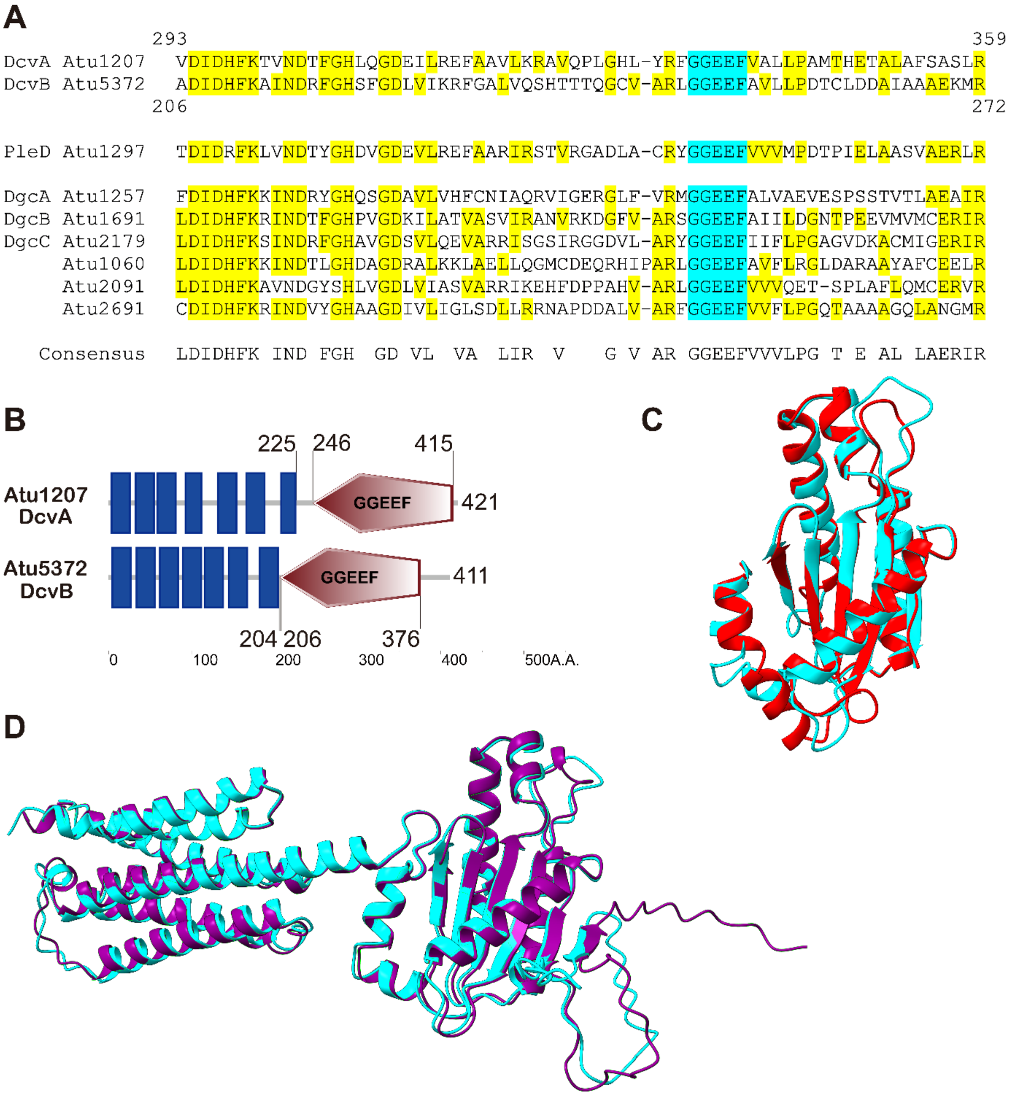

Fig. S7

Overexpression of catalytic inactive DcvB loses its regulation on planktonic cell growth and induces cell aggregation.

(A) The planktonic cell density measured by optical density (O.D.$_{600}$) with strains overexpressing wild-type DcvB or catalytic mutant while culturing in AS induction medium for 8 hours. The solid line indicates the presence of IPTG induction, and the dashed line represents the absence of IPTG for low level expression. (B) Biofilm formation stained by crystal violet and quantified by absorbance value (A_{595}) with strains overexpressing wild-type DcvB or catalytic mutant. Data are one representative result with mean ± SD of three biological replicates. Comparable results were observed in two independent experiments. The number atop each bar represents the *P*-value of one-way ANOVA with Fisher's LSD test obtained by comparing to the vector control. The term 'ns' denotes no significant difference (*P*>0.01).

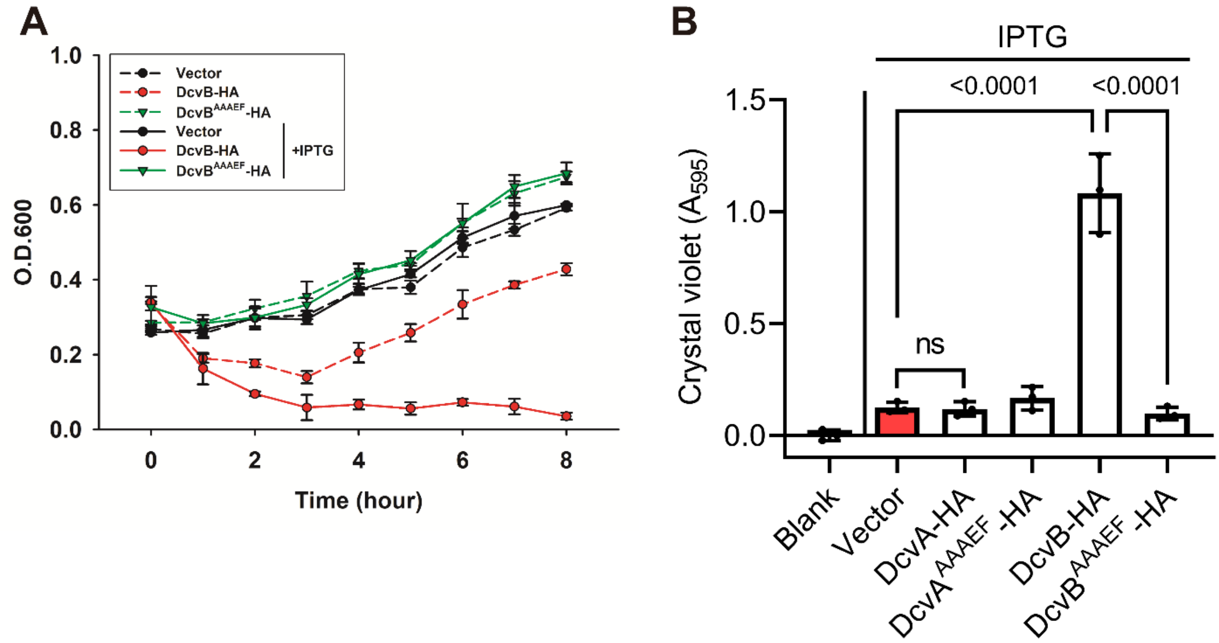

Fig. S8

DcvA inhibits virulence protein accumulation with its transmembrane region.

Accumulation of virulence proteins in strains overexpressing DcvA variants after AS induction for 24 hours. "AS-" is included as non-induced control with the addition of DMSO. α-RpoA is included as an internal control, α-HA is included to detect the overexpression of HA-tagged DcvA variants. The red arrow on the blotting results indicates the potential signal of DcvA variants; the blue square on the sequence line indicates the transmembrane region. High molecular weight protein signals from all DcvA variants containing intact or most of N-terminal transmembrane region but not from DcvA^{DGC}, suggesting the oligomerization of DcvA via transmembrane region.

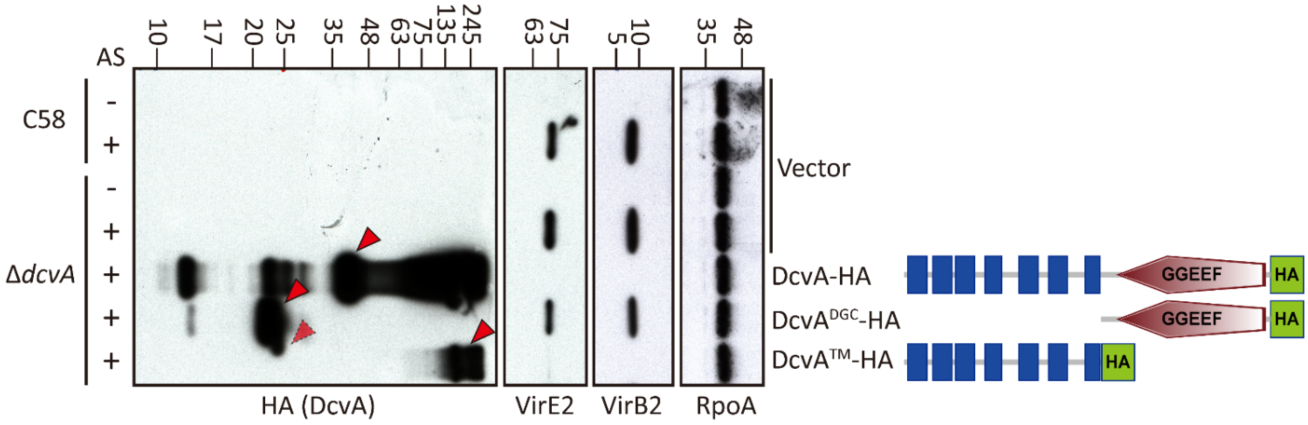

Fig. S9

Hypothetical regulatory model of DGC via two-component systems.

(A) Schematic of the regulation of the VirA/VirG two-component system by DGC. Phenolic AS activates VirA, which then phosphorylates VirG, leading to virulence gene transcription. The phosphomimic VirG^{N54D} mutant constitutively activates virulence gene transcription without VirA. If no virulence transcription is observed, DGC likely targets downstream of VirA, potentially VirG; If transcription remains, the target is likely upstream of VirG, such as VirA or ChvG/ChvI. (B) Schematic of the regulation of the ChvG/ChvI two-component system by DGC. An acidic environment activates ChvG, leading to ChvI phosphorylation and T6SS gene activation. The phosphomimic ChvI^{D52E} mutant constitutively activates T6SS gene transcription without ChvG. If no T6SS transcription is observed, DGC likely targets downstream of ChvG, potentially ChvI; If transcription remains, the target is likely upstream of ChvI, such as ChvG or other undetermined pathways.

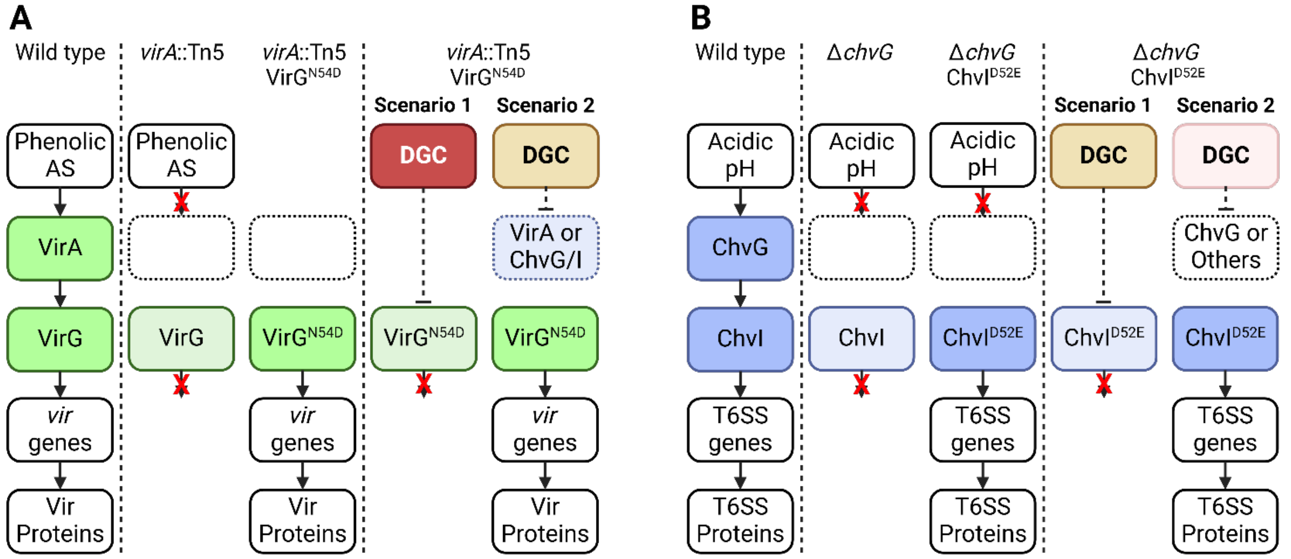

Fig. S10

Overexpression of DcvA inhibits virulence protein accumulation in phosphomimic VirG^{N54D} strain.

Accumulation of virulence protein in phosphomimic VirG^{N54D} strains after culturing in acidic minimal medium for 24 hours. Phenolic AS was added for virulence induction, and DMSO was added as non-induced control. C58 and its derived virulent strain T-DNA::Tn5 are positive controls of AS induction for comparison to *vir* induction level while constitutively expressing VirG^{N54D} in *virA* mutant (*virA*::Tn5) (3). The HA-tagged DGCs and VirG^{N54D} were expressed using two different plasmid, pTrc200 and pRLop, respectively. α-RpoA is included as an internal control, α-HA is included to detect the overexpression of HA-tagged DGC. Comparable results were observed in two independent experiments.

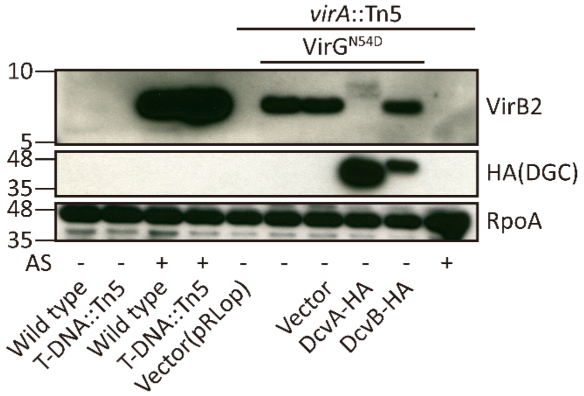

Fig. S11

DcvA and DcvB exhibit similar secondary structure and conserved DGC domain.

(A) The alignment of full-length amino acid sequences of DcvA and DcvB. The number at each site's corner indicates its amino acid position of the protein. Identical amino acids between the sequences are indicated, and "+" denotes similar amino acids. Black wave lines represent α-helices, arrows indicate β-sheets, red wave lines highlight predicted transmembrane (TM) regions and shaded light blue region marks predicted DGC domains. The identity and similarity between amino acids are 23.77%/40.69% for full length, 19.18%/35.10% for the N-terminal transmembrane region, and 34.08%/54.75% for the DGC domain. The α-helices 0-4 and β-sheets 1-6 are labeled based on the common core fold described in reference (4).

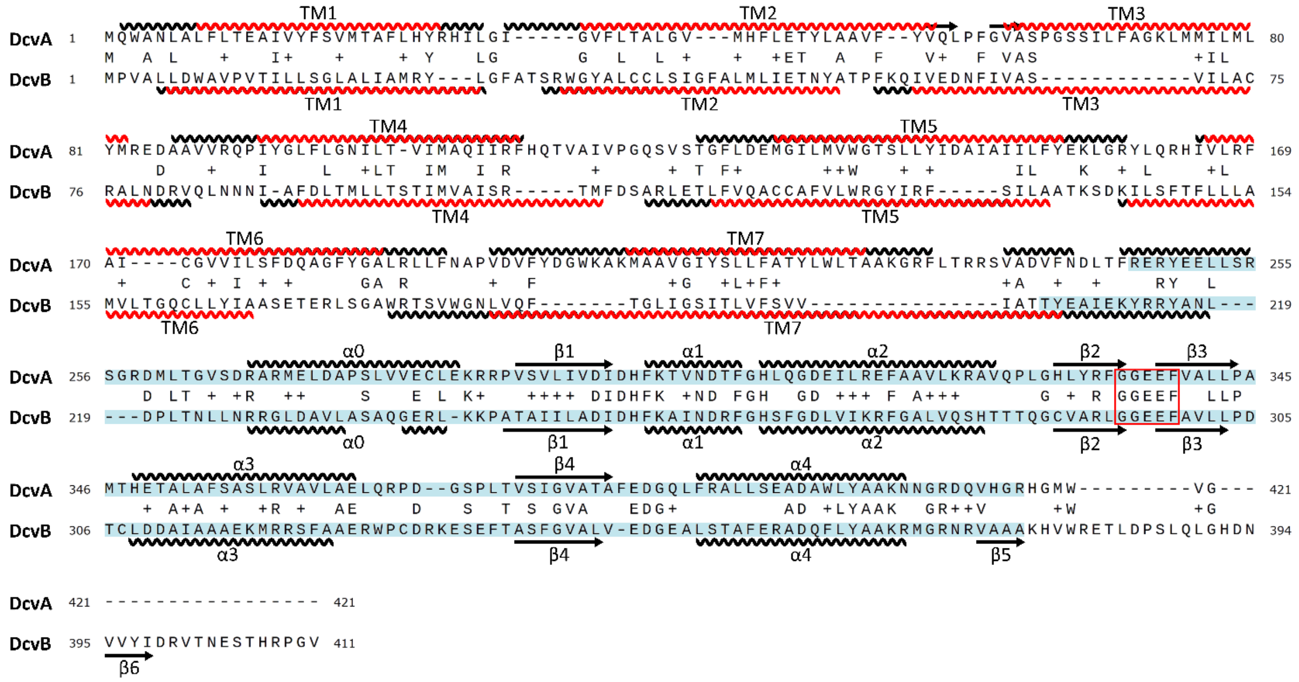

Table 1. Strains used in this study.

Bacterial strains	Relevant characteristics	References	Lab No.
***E. coli* strain**			
DH10B	strain K-12 F– *mcrA* Δ(*mrr-hsdRMS-mcrBC*) φ80*lacZ*ΔM15 Δ*lacX74 recA1 endA1 araD139* Δ (*ara-leu*)7697 *galU galK* λ– *rpsL*(Str^R) *nupG*, for construction.	Invitrogen	EML455
BW25113	strain K-12 *lacI*$^{+}$*rrnB*$_{T14}$ Δ*lacZ*$_{WJ16}$ *hsdR*514 Δ*araBAD*$_{AH33}$ Δ*rhaBAD*$_{LD78}$ *rph-1* Δ*(araB–D)567* Δ*(rhaD–B)568* Δ*lacZ4787*(::*rrnB-3*) *hsdR514 rph-1*, for interbacterial competition.	(5)	EML5398
***Agrobacterium* strains**			
C58	Wild type virulent strain containing nopaline-type Ti plasmid pTiC58.	E. W. Nester's Lab	EML530
C58 Δ*dcvA*	*dcvA* (*atu1207*) deletion mutant in C58.	This study	EML2399
C58 Δ*dcvB*	*dcvB* (*atu5372*) deletion mutant in C58.	This study	EML2400
C58 Δ*dcvA*Δ*dcvB*	*dcvA* and *dcvB* double deletion mutant.	This study	EML2401
C58 Δ*virB2*	*virB2* deletion mutant in C58.	(6)	EML2254
C58 Δ*tssM*	*tssM* deletion mutant in C58.	(7)	EML1068
C58 Δ*tssL*	*tssL* deletion mutant in C58.	(7)	EML1073
C58 Δ*chvG*	*chvG* deletion mutant in C58.	(8)	EML1226
NT1RE	C58 cured of its pTiC58 (Ti plasmid^{-}).	(9)	EML6
T-DNA::Tn5	Km^R, NT1RE containing pJK270 plasmid, which is pTiC58^{TraC} with Tn5 insertion in the T-DNA region without affecting virulence.	(10)	EML7
virA::Tn5	Km^R, NT1RE containing pJK107 plasmid, which is pTiC58^{TraC} with Tn5 insertion in *virA*.	(10)	EML142

Table 2. Plasmids used in this study.

Plasmids	Relevant characteristics	References	Lab No.
**Expression plasmids**			
pET-28a(+)	Km^R, *E. coli* overexpression vector to produce N or C-terminal His-tagged protein.	Novagen	EML2485
pTrc200	Sp^R, pVS1 origin *lacIq*, *trc* (fusion of *trp* and *lac*) promoter expression vector.	(11)	EML904
pDcvA-HA	Sp^R, *dcvA* gene encoding DcvA with C-terminal HA tag was cloned into pTrc200 vector for overexpressing DcvA-HA.	(12)	EML2381
pDcvA^{AAAEF}-HA	Sp^R, *dcvA* gene encoding DcvA with the GGEEF motif substituted by AAAEF and tagged with C-terminal HA was cloned into the pTrc200 vector for overexpressing DcvA^{AAAEF}-HA.	This study	EML2382
pDcvA^{DGC}-HA	Sp^R, cytosolic DGC domain of *dcvA* gene tagged with C-terminal HA was cloned into the pTrc200 vector for overexpressing DcvA^{DGC}-HA.	This study	EML4985
pDcvA^{TM}-HA	Sp^R, transmembrane domain of *dcvA* gene tagged with C-terminal HA was cloned into the pTrc200 vector for overexpressing DcvA^{TM}-HA.	This study	EML4986
pDcvB-HA	Sp^R, *dcvB* gene encoding DcvB with C-terminal HA tag was cloned into pTrc200 vector for overexpressing DcvB-HA.	(12)	EML2383
pDcvB^{AAAEF}-HA	Sp^R, *dcvB* gene encoding DcvB with the GGEEF motif substituted by AAAEF and tagged with C-terminal HA was cloned into the pTrc200 vector for overexpressing DcvB^{AAAEF}-HA.	This study	EML2384
pRL662	Gm^R, broad host range plasmid derived from pBBR1-MCS2, in which Km^R, *mob* and *oriT* were replaced by a Gm^R marker. The *lac* promoter can be used for constitutively expressing gene of interest in the absence of *lacI* repressor gene.	(13)	EML315
pRLop	Gm^R, *lac* operator was removed from pRL662 plasmid for constitutively expressing gene of interest even in the presence of *lacI* repressor gene.	This study	EML2394
pVirG^{N54D}	Gm^R, phosphomimic *virG*^{N54D} mutant was cloned into pRLop vector for expressing VirG^{N54D} driven by constitutively active *lac* promoter.	This study	EML2396

pChvI^{D52E}	Gm^R, phosphomimic *chvI*^{D52E} mutant was cloned into pRL662 vector for expressing ChvI^{D52E} driven by constitutively active *lac* promoter.	(8)	EML3130
**Suicide plasmids**			
pJQ200ks	Gm^R, pJQ200ks was derived from pJQ200, which contains *sacB* from pUCD800 via pUM24. It includes Gm^R *traJ oriT* for the selection of double crossover events.	(14)	EML1432
pJQ*1207*UD	Gm^R, the pJQ200ks vector carries the fusion of 500-bp upstream and 500-bp downstream sequence from *dcvA* gene, used to generate *dcvA* deletion mutant.	This study	
pJQ*5372*UD	Gm^R, the pJQ200ks vector carries the fusion of 500-bp upstream and 500-bp downstream sequence from *dcvB* gene, used to generate *dcvB* deletion mutant.	This study	EML2392
**Reporter plasmids**			
pBISN1	Km^R, binary vector expressing *gusA*-intron driven by super promoter.	(15)	EML308
pRU1156	Ap^R, a stable broad host range promoter-probe vector containing *gfpmut3.1* and *gusA*.	(16)	EML579
pRU*virB*p	Ap^R, the promoter region of *virB* was cloned into pRU1156 in the upstream of promoter-less *gfp* to generate *virB*$_{promoter}$:*gfp-gus* transcriptional fusion.	(17)	EML659
pRU*virE*p	Ap^R, the promoter region of *virE* was cloned into pRU1156 in the upstream of promoter-less *gfp* to generate *virB*$_{promoter}$:*gfp-gus* transcriptional fusion.	(18)	EML1202

Table 3. Primers used in this study.

Primers	Sequence (5'-3')	R.E. site	References
**For cloning on expression plasmids**			
dcvA-F	AAAAACCATGGACATGCAATGGGCAAATCTGGCCCTC	NcoI	(12)
dcvA-R	AAAAATCTAGAGCCGACCCACATGCCATGACG	XbaI	(12)
dcvA-AAAEF-F	CTGGGACATCTTTACCGTTTCGCCGCCGCGGAATTCGTCGCGCTCCTGCCG		This study
dcvA-AAAEF-R	CGGCAGGAGCGCGACGAATTCCGCGGCGGCGAAACGGTAAAGATGTCCCAG		This study
dcvA-cyto-DF	AAAAACCATGGACATGAAGGGCCGCTTTCTGAC	NcoI	This study
dcvA-TM-DR	AAAAATCTAGAGGCCGCTGTCAGCCAGAGAT	XbaI	This study
dcvB-F	AAAAACCATGGACATGCCTGTAGCTTTACTTGATTGGGCA	NcoI	(12)
dcvB-R	AAAAATCTAGAAACGCCCGGTCGGTGGGTACT	XbaI	(12)
dcvB-AAAEF-F	AAGGCTGCGTTGCTCGCCTAGCCGCTGCGGAGTTTGCGGTCCTCCTTCC		This study
dcvB-AAAEF-R	GGAAGGAGGACCGCAAACTCCGCAGCGGCTAGGCGAGCAACGCAGCCTT		This study
pRL662-Δoperator-F	ATTCCACAGCCTGGGGTGCCTAATGAG		This study
pRL662-Δoperator-R	CCCAGGCTGTGGAATTTCACACAGGAA		This study
virG-F	AAAAAGAATTCTGTATAAATTCTGTTGAGCTGCAAATGG	EcoRI	This study
virG-R	AAAAATCTAGATCAGGCCGCCATCACACCCCCGTA	XbaI	This study
virG-N54D-F	GTCGTGGTCGTCGATCTTGATTTGGGTCGCGAAGATGGG		This study
virG-N54D-R	CCCATCTTCGCGACCCAAATCAAGATCGACGACCACGAC		This study
**For generating *dgc* mutants**			
dcvA-UF	AAAAAACTAGTCTCCGGCCAGCCGTCTTCCGACGCGCC	SpeI	This study
dcvA-UR	ATCGCCGGCATCAGCCGACCCACATCCATTGCATCGGCACACGCCCTATC		This study
dcvA-DF	GATAGGGCGTGTGCCGATGCAATGGATGTGGGTCGGCTGATGCCGGCGAT		This study
dcvA-DR	AAAAACTCGAGCGATCCGCAAGCCCGCCGACCAGCGCAC	XhoI	This study
dcvB-UF	AAAAATCTAGATCACGACGGAGCCGCTCGATCTCAA	XbaI	This study

dcvB-UR	AGCTTTAGTCTCATCCTCAAACGCCTACAGGCATAAAGGCTTGAGTGCTC		This study
dcvB-DF	GAGCACTCAAGCCTTTATGCCTGTAGGCGTTTGAGGATGAGACTAAAGCT		This study
dcvB-DR	AAAAACTCGAGCTCCGGCTCAGATCGAAGCCTAATA	XhoI	This study
**For RT-qPCR**			
16s-F	TGGAGCATGTGGTTTAATTCGA		(19)
16s-R	TGCGGGACTTAACCCAACA		(19)
C58-*virB2*-RTqPCR-F	ATCTGAATCGCCTCTCGCTCTCGAA		This study
C58-*virB2*-RTqPCR-R	TTAACCATTGTGGCTGGGTCAGTGC		This study
C58-*virE2*-RTqPCR-F	TCACTGAGACCGCAGCAGGCAATGT		This study
C58-*virE2*-RTqPCR-R	CGGGCTTCCGTGCATGTTGTGTTCG		This study
C58-*virG*-RTqPCR-F	GTTGGGAGCAACCGATTTTA		This study
C58-*virG*-RTqPCR-R	TTTCACCTCACTGCCCTCTT		This study
C58-*tssM*-RTqPCR-F	AGGAGCAGAATGCGGAAATA		This study
C58-*tssM*-RTqPCR-R	GGGAAGATGACGTCCTTGAA		This study
C58-*hcp*-RTqPCR-F	CCACAAAGCTTTTCCAGGAG		This study
C58-*hcp*-RTqPCR-R	ACGGCGTGTACTTCACTTCC		This study

Reference for Materials

1. Xu J, Kim J, Koestler BJ, Choi JH, Waters CM, Fuqua C. 2013. Genetic analysis of *Agrobacterium tumefaciens* unipolar polysaccharide production reveals complex integrated control of the motile-to-sessile switch. Molecular Microbiology 89:929-948.
2. Letunic I, Khedkar S, Bork P. 2021. SMART: recent updates, new developments and status in 2020. Nucleic Acids Res 49:D458-D460.
3. Rogowsky PM, Close TJ, Chimera JA, Shaw JJ, Kado CI. 1987. Regulation of the *vir* genes of *Agrobacterium tumefaciens* plasmid pTiC58. J Bacteriol 169:5101-12.
4. Schirmer T. 2016. C-di-GMP synthesis: structural aspects of evolution, catalysis and regulation. J Mol Biol 428:3683-701.
5. Datsenko KA, Wanner BL. 2000. One-step inactivation of chromosomal genes in *Escherichia coli* K-12 using PCR products. Proc Natl Acad Sci U S A 97:6640-5.
6. Wu HY, Chen CY, Lai EM. 2014. Expression and functional characterization of the *Agrobacterium* VirB2 amino acid substitution variants in T-pilus biogenesis, virulence, and transient transformation efficiency. PLoS One 9:e101142.
7. Ma LS, Lin JS, Lai EM. 2009. An IcmF family protein, ImpLM, is an integral inner membrane protein interacting with ImpKL, and its walker a motif is required for type VI secretion system-mediated Hcp secretion in *Agrobacterium tumefaciens*. J Bacteriol 191:4316-29.
8. Wu CF, Lin JS, Shaw GC, Lai EM. 2012. Acid-induced type VI secretion system is regulated by ExoR-ChvG/ChvI signaling cascade in *Agrobacterium tumefaciens*. PLoS Pathog 8:e1002938.
9. Watson B, Currier TC, Gordon MP, Chilton MD, Nester EW. 1975. Plasmid required for virulence of *Agrobacterium tumefaciens*. J Bacteriol 123:255-64.
10. Kao JC, Perry KL, Kado CI. 1982. Indoleacetic acid complementation and its relation to host range specifying genes on the Ti plasmid of *Agrobacterium tumefaciens*. Mol Gen Genet 188:425-32.
11. Schmidt-Eisenlohr H, Domke N, Baron C. 1999. TraC of IncN plasmid pKM101 associates with membranes and extracellular high-molecular-weight structures in *Escherichia coli*. J Bacteriol 181:5563-71.
12. McCarthy RR, Yu M, Eilers K, Wang YC, Lai EM, Filloux A. 2019. Cyclic di-GMP inactivates T6SS and T4SS activity in *Agrobacterium tumefaciens*. Mol Microbiol 112:632-648.
13. Vergunst AC, Schrammeijer B, den Dulk-Ras A, de Vlaam CM, Regensburg-Tuink TJ, Hooykaas PJ. 2000. VirB/D4-dependent protein translocation from *Agrobacterium* into plant cells. Science 290:979-82.
14. Quandt J, Hynes MF. 1993. Versatile suicide vectors which allow direct selection for gene replacement in gram-negative bacteria. Gene 127:15-21.

15. Narasimhulu SB, Deng XB, Sarria R, Gelvin SB. 1996. Early transcription of *Agrobacterium* T-DNA genes in tobacco and maize. Plant Cell 8:873-86.

16. Karunakaran R, Mauchline TH, Hosie AHF, Poole PS. 2005. A family of promoter probe vectors incorporating autofluorescent and chromogenic reporter proteins for studying gene expression in Gram-negative bacteria. Microbiology (Reading) 151:3249-3256.

17. Tsai YL, Wang MH, Gao C, Klusener S, Baron C, Narberhaus F, Lai EM. 2009. Small heat-shock protein HspL is induced by VirB protein(s) and promotes VirB/D4-mediated DNA transfer in *Agrobacterium tumefaciens*. Microbiology (Reading) 155:3270-3280.

18. Hwang HH, Yang FJ, Cheng TF, Chen YC, Lee YL, Tsai YL, Lai EM. 2013. The Tzs protein and exogenous cytokinin affect virulence gene expression and bacterial growth of *Agrobacterium tumefaciens*. Phytopathology 103:888-99.

19. Yang S, Lin S, Kelen GD, Quinn TC, Dick JD, Gaydos CA, Rothman RE. 2002. Quantitative multiprobe PCR assay for simultaneous detection and identification to species level of bacterial pathogens. J Clin Microbiol 40:3449-54.
